# Qualitative metabolomics profiling of serum and bile from dogs with gallbladder mucocele formation

**DOI:** 10.1371/journal.pone.0191076

**Published:** 2018-01-11

**Authors:** Jody L. Gookin, Kyle G. Mathews, John Cullen, Gabriela Seiler

**Affiliations:** 1 Department of Clinical Sciences, College of Veterinary Medicine and Comparative Medicine Institute, North Carolina State University, Raleigh, North Carolina, United States of America; 2 Department of Population Health and Pathobiology, College of Veterinary Medicine and Comparative Medicine Institute, North Carolina State University, Raleigh, North Carolina, United States of America; 3 Department of Molecular Biomedical Sciences, College of Veterinary Medicine and Comparative Medicine Institute, North Carolina State University, Raleigh, North Carolina, United States of America; University of Illinois, UNITED STATES

## Abstract

Mucocele formation is characterized by secretion of abnormally thick mucus by the gallbladder epithelium of dogs that may cause obstruction of the bile duct or rupture of the gallbladder. The disease is increasingly recognized and is associated with a high morbidity and mortality. The cause of gallbladder mucocele formation in dogs is unknown. There is a strong breed predisposition and affected dogs have a high incidence of concurrent endocrinopathy or hyperlipidemia. These observations suggest a significant influence of both genetic and metabolic factors on disease pathogenesis. In this study, we investigated a theory that mucocele formation is associated with a syndrome of metabolic disruption. We surmised that a global, untargeted metabolomics approach could provide unique insight into the systemic pathogenesis of gallbladder mucocele formation and identify specific compounds as candidate biomarkers or treatment targets. Moreover, concurrent examination of the serum and hepatic duct bile metabolome would enable the construction of mechanism-based theories or identification of specific compounds responsible for altered function of the gallbladder epithelium. Abnormalities observed in dogs with gallbladder mucocele formation, including a 33-fold decrease in serum adenosine 5’-monophosphate (AMP), lower quantities of precursors required for synthesis of energy transporting nucleotides, and increases in citric acid cycle intermediates, suggest excess metabolic energy and a carbon surplus. Altered quantities of compounds involved in protein translation and RNA turnover, together with accumulation of gamma-glutamylated and N-acetylated amino acids in serum suggest abnormal regulation of protein and amino acid metabolism. Increases in lathosterol and 7α-hydroxycholesterol suggest a primary increase in cholesterol synthesis and diversion to bile acid formation. A number of specific biomarker compounds were identified for their ability to distinguish between control dogs and those that formed a gallbladder mucocele. Particularly noteworthy was a significant decrease in quantity of biologically active compounds that stimulate biliary ductal fluid secretion including adenosine, cAMP, taurolithocholic acid, and taurocholic acid. These findings support the presence of significant metabolic disruption in dogs with mucocele formation. A targeted, quantitative analysis of the identified serum biomarkers is warranted to determine their utility for diagnosis of this disease. Finally, repletion of compounds whose biological activity normally promotes biliary ductal secretion should be examined for any therapeutic impact for resolution or prevention of mucocele formation.

## Introduction

Formation of bile is a unique function of the liver and is essential for survival. Secretion of bile solutes begins at the canalicular membrane of hepatocytes where a diverse collection of membrane proteins mediate transport of functional products, metabolic waste, and exogenous xenobiotics into the biliary canaliculi. Many transporters are members of the ATP-binding cassette (ABC) superfamily with responsibilities for energy-dependent secretion of specific substances such as bile acids, glutathione, cholesterol, phospholipids, and a myriad of other lipophilic and conjugated substances into bile. Bile is secreted into a ductular system lined by biliary epithelial cells whose maintenance of fluidity, pH, and ion composition is critical to ensuring bile flow, preventing precipitation of solutes, and promoting hydrophilicity of bile acids[1]. These functions are controlled by exogenous hormones such as secretin, as well as bile constituents such as nucleotides and bile acids that regulate the activity of specific epithelial ion transport proteins. In most species, bile is temporarily stored in the gallbladder and later discharged into the intestinal tract when food intake promotes cholecystokinin-induced gallbladder contraction. In addition to providing a physical barrier for containment of bile, the gallbladder epithelium plays a complex role in reabsorption of water and electrolytes, reclamation of bile cholesterol, lipids, amino acids, and bile acids, and participates in the metabolism and extrusion of xeno- and endobiotics[2]. The integrity of the gallbladder epithelium and its functions are protected by a blanket of secreted mucus and bicarbonate that serves as a barrier against exposure to toxic components of bile.

Gallbladder mucocele formation is an emergent disease in dogs[3–13]. The disease is characterized by a relentless secretion of abnormally thick mucus by the gallbladder epithelium that can result in obstruction of the bile duct or rupture of the gallbladder. Despite surgery to remove the gallbladder, a median of 27% of dogs (range, 7 to 45%) die or are euthanized within 2 weeks of hospitalization[5, 7, 9, 11–14]. The cause of gallbladder mucocele formation in dogs is unknown. Theories that poor gallbladder motility, gallbladder obstruction, or biliary infection are causative of gallbladder mucocele formation are not supported by clinical or experimental evidence in dogs[5, 7, 9, 11–16]. Efforts to link a genetic defect in ATP Binding Cassette Subfamily B Member 4 (ABCB4), a hepatocyte canicular membrane phosphatidylcholine floppase, was initially promising but later unsupported[17]. Gallbladder mucocele formation is diagnosed in older-aged (median, 10 years) pure breeds of dog such as the Shetland sheepdog, Cocker spaniel, Miniature Schnauzer, Pomeranian, Chihuahua, and others[5, 7, 14, 18, 19]. Two cases have been reported in cats[20, 21]. Affected dogs have a high incidence of concurrent endocrinopathy in the form of hypothyroidism or hyperadrenocorticism[5, 10, 18, 19] and a variable concurrence of hypercholesterolemia or hypertriglyceridemia[5, 18]. These observations suggest a significant influence of both genetic and metabolic factors on disease pathogenesis.

As a basis for better understanding the cause of abnormal mucus accretion, recent studies of normal and affected gallbladders identified that mucocele formation is associated with excess secretion of Muc5ac, a gel-forming mucin, by the gallbladder epithelium[22]. Muc5ac is highly cross-linked and entangled by a specific repertoire of mucin-interacting proteins. During exocytosis, mucus granules fail to unpack their contents or break free from the vesicles and remain tethered to one another and to the gallbladder epithelium[22]. This observation is highly reminiscent of the properties of mucus in the setting of cystic fibrosis[23, 24] where a failure to maintain the fluid, ionic, and acid-base composition of the mucosal surface instigates immobilization and adhesion of mucus to the epithelium. In fact, studies of newborn pigs and juvenile ferrets with cystic fibrosis are characterized by mucus accumulation[25] and cystic mucosal hypertrophy of the gallbladder[26] that appear nearly identical to gallbladder mucoceles from dogs.

In this study, we explored an overall theory that gallbladder mucocele formation is related to an acquired metabolic-type syndrome that is capable of altering secretory function of the gallbladder epithelium in genetically-predisposed breeds of dog. Accordingly, we performed a global untargeted metabolomics characterization of the serum and nascent hepatic duct bile of dogs with mucocele formation compared to control dogs. Our rationale was that a characterization of abnormal metabolic pathways would provide key insights into the pathogenesis of gallbladder mucocele formation in dogs and identify mechanistic causes for abnormal gallbladder epithelial function.

## Methods

### Patient and control dog populations

Dogs that were diagnosed with a gallbladder mucocele at the North Carolina State University Veterinary Hospital (NCSU-VH) and undergoing surgical removal of the gallbladder were prospectively identified on the basis of supportive findings of gallbladder ultrasonography as previously described[22]. Dogs were included in the study after obtaining informed owner consent. Control dogs were purpose-bred research animals that were prospectively identified as having no history of gastrointestinal or hepatobiliary disease and were not receiving any medications. Control dogs were scheduled to undergo euthanasia for purposes of colony depopulation unrelated to this study at which time tissues became available for collection. All animal use was carried out in accordance with the recommendations in the Guide for the Care and Use of Laboratory Animals of the United States National Institutes of Health. The protocol was approved by the Institutional Animal Care and Use Committee of North Carolina State University.

### Sample collections

Serum from dogs with mucocele formation was obtained from non-heparinized whole blood samples that were collected ≤ 36 hours prior to surgery and submitted to the Clinical Pathology Laboratory of the NCSU-VH where serum was separated by means of centrifugation at 2150 × g for 10 min at 4°C. Serum biochemical analysis was performed using a Roche Cobas c501 chemistry analyzer and the leftover serum was stored in the Clinical Pathology Laboratory at 4°C for less than 72 hours before retrieval by study investigators for storage at -80°C. Duration of fasting prior to blood sample collection in dogs with mucocele formation was not known. Blood samples from control dogs were collected under sedation immediately prior to intravenous administration of a lethal dose of pentobarbital solution. Serum was separated as previously described and immediately frozen at -80°C. All control dogs were fasted a minimum of 12 hours prior to blood sample collection.

Bile samples from dogs with mucocele formation were obtained intra-operatively. Following surgical removal of the gallbladder, a single sample of bile was collected from an hepatic duct by inserting a sterile 3.5 French red rubber catheter retrograde through the severed end of the cystic duct. In dogs undergoing concurrent duodenotomy, hepatic duct bile was collected by passage of the catheter through the bile duct into an hepatic duct via the duodenal papilla. A swab sample of gallbladder content was collected intraoperatively by the attending surgeon and submitted to the NCSU-VH Clinical Microbiology laboratory for aerobic and anaerobic bacterial culture as previously described[27]. Gallbladder tissue was placed into formalin by the attending surgery technician and submitted to the NCSU-VH Histopathology laboratory for routine processing for light microscopic examination by the on-duty veterinary pathologist.

For control dogs, bile samples were obtained immediately after euthanasia via midline laparotomy followed by fine needle (22 gauge) aspiration from an hepatic duct. Bile and serum samples were stored in 1.6 to 2 milliliter microcentrifuge tubes at -80°C for up to 20 months prior to batch analysis. Gallbladder tissue was placed into formalin by the study investigators and submitted to the NCSU-VH Histopathology laboratory for routine processing for light microscopic examination. Bacterial culture of bile from control dogs was not performed.

### Collection of medical record data

Information recorded from the medical record of dogs with gallbladder mucocele formation included age, breed, sex, results of pre-operative complete blood cell count and serum biochemical analysis, ultrasonographic findings pertaining to the gallbladder, gross and histopathological description of the gallbladder, results of aerobic and anaerobic bacterial culture of gallbladder contents. For control dogs recorded medical record data included age, breed, and sex. A list of all drugs and chemicals to which each dog was known to be exposed at the time of sample collection was recorded.

### Criteria for selection of cases for mass spectrometric analysis

A target sample size of 10 dogs with gallbladder mucocele formation and 10 control dogs was sought prospectively. Selection criteria for dogs to be included in the study were 1) availability of matching serum and bile samples, 2) sufficient quantity and quality of hepatic duct bile for mass spectrometric analysis, 3) a serum total bilirubin ≤ 2.5 mg/dl, and 4) gross and light microscopic confirmation of gallbladder mucocele formation or of a normal gallbladder (control dogs). Dogs having serum bilirubin > 2.5 mg/dl were excluded from the study in an effort to minimize the impact of overt cholestasis on compounds detected by mass spectrometric analysis.

### Mass spectrometry and compound identification

Samples were extracted and analyzed on GC/MS and UPLC-MS/MS platforms by a commercial laboratory (Metabolon Inc., Durham, NC). Sample processing, data extraction, compound identification, and curation are described in Supplementary Methods (S1 Methods).

### Classification analysis

Random forest analysis[28] was performed using the program “R” (http://cran.r-project.org/) to identify individual compounds that were best able to predict disease classification (i.e. presence of mucocele formation). Compounds contributing the most to the accuracy of disease classification were identified as those having the highest mean decrease accuracy (MDA) of the predicted classification when permuted within the dataset.

### Statistical analysis

Instrument and processing variability were determined by calculating the median relative standard deviation for internal standards that were added to each sample and for all endogenous metabolites present in 100% of pooled sample replicates. Data were log transformed and missing values, if any, were imputed with the minimum observed value for each compound. Welch’s two-sample t-test was used to identify biochemicals that differed significantly between experimental groups. An estimate of the false discovery rate (q-value)[29] was calculated to take into account the effect of multiple comparisons. A low q-value (q<0.10) is an indication of high confidence while a higher q-value indicates diminished confidence of a result.

## Results

### Selection criteria of case and control dogs

Twenty one dogs diagnosed with gallbladder mucocele formation based on ultrasonographic findings and undergoing surgical removal of the gallbladder were enrolled in the study. Ten dogs had samples selected for inclusion in mass spectrometric analysis. Dogs not selected for inclusion had insufficient quantity of hepatic duct bile obtained for analysis (n = 5), observation of gross contamination of bile with blood at the time of collection (n = 3), equivocal diagnosis of gallbladder mucocele formation based on histopathological findings (n = 2), and no concurrent serum sample available (n = 1).

Twenty four control dogs were enrolled in the study. Ten dogs had samples selected for inclusion in mass spectrometric analysis. Dogs not selected for inclusion had insufficient quantity of bile obtained for analysis (n = 7), observation of gross contamination of bile with blood at the time of collection (n = 2), and grossly abnormal appearing gallbladder content (n = 1). The remaining 4 dogs were not chosen based on not having been fasted prior to euthanasia (n = 2), concurrent administration of an antibiotic for pyoderma (n = 1), and no reason other than that the dog was not needed (n = 1).

### Description of case and control dogs

Breeds of dog with gallbladder mucocele formation included in the study were Shetland sheepdog (n = 3), American Cocker Spaniel (n = 2) and English Cocker Spaniel, Chihuahua, Beagle, Shih Tzu, and Italian Greyhound (n = 1 each). The median age was 11.4 years (range, 1.9 to 15.2 years). Four dogs were ovariohysterectomized females, four dogs were castrated males, and two dogs were reproductively intact females. Hepatic duct bile was collected from the severed cystic duct in 8 dogs and via duodenotomy with catheterization of an hepatic duct via the common bile duct in 2 dogs. Recorded drug and chemical exposures at the time of serum or bile collection from dogs with mucocele formation are shown in S1 Table.

Breeds of control dogs included Beagle (n = 8) and mongrel (n = 2). The median age was 5.9 years (range, 1.9 to 7.3 years). Eight dogs were reproductively intact males and two dogs were ovariohysterectomized females. Hepatic duct bile was collected from each dog by direct fine needle aspiration. In all dogs a normal gallbladder was documented by gross examination and histologically.

### Laboratory and gallbladder pathology findings

All 10 dogs diagnosed with gallbladder mucocele formation had a complete blood cell count and serum biochemical analysis performed prior to surgery. As shown in Table 1, the most common abnormality identified on the complete blood cell count was a mild to moderate neutrophilia and the presence of immature (band) neutrophils. The most common abnormalities observed on serum biochemical analysis were increases in alkaline phosphatase, alanine transaminase and gamma-glutamyltransferase enzyme activities, which were above the reference range in ≥ 80% of dogs. Only 3 dogs with gallbladder mucocele formation had a total bilirubin above the laboratory reference range and none were clinically icteric.

**Table 1:** Pre-operative complete blood cell count and serum biochemistry variables pertinent to inflammation and cholestasis in whole blood and serum, respectively, from 10 dogs diagnosed with a gallbladder mucocele.

Clinical Pathological Variable	Median	Range	Number (%) of dogs with value outside reference range	Reference range
**Complete blood cell count**				
Packed cell volume (%)	49	37–51	3/10 (30)	39–58
Plasma protein (g/dl)	7.5	6.2–10	5/10 (50)	6.1–7.5
Neutrophils (× 10^3^/μl)	10.158	7.199–22.091	5/10 (50)	2.841–9.112
Immature (band) neutrophils (× 10^3^/μl)	0.109	0–2.530	7/10 (70)	0.0–0.0
Platelets (× 10^3^/μl)	437	309–937	3/10 (30)	191–468
**Serum biochemical analysis**				
ALP (IU/L)	850	24–2545	8/10 (80)	16–140
ALT (IU/L)	270	20–1403	9/10 (90)	12–54
GGT (IU/L)	23	0–282	8/10 (80)	0–6
Total bilirubin (mg/dl)	0.1	0.1–2.3	3/10 (30)	0–0.2
Cholesterol (mg/dl)	316	152–875	4/10 (40)	124–344
Lipase (IU/L)	129	16–189	2/10 (20)	12–147
Amylase (IU/L)	645	281–1397	2/10 (20)	236–1337

ALP, alkaline phosphatase; ALT, alanine transaminase; GGT, gamma-glutamyltransferase

All 10 dogs had a sample of gallbladder content submitted for both aerobic and anaerobic bacterial culture. Two dogs were positive for aerobic growth of an alpha-hemolytic *Streptococcus* sp. (in thioglycolate broth only) and *Streptococcus gallolyticus*, respectively. An additional dog was aerobic culture positive for the presence of an alpha-hemolytic *Streptococcus* sp. (in thioglycolate broth only) on a sample of liver tissue that was submitted at the discretion of the attending surgeon.

**Fig 1:**
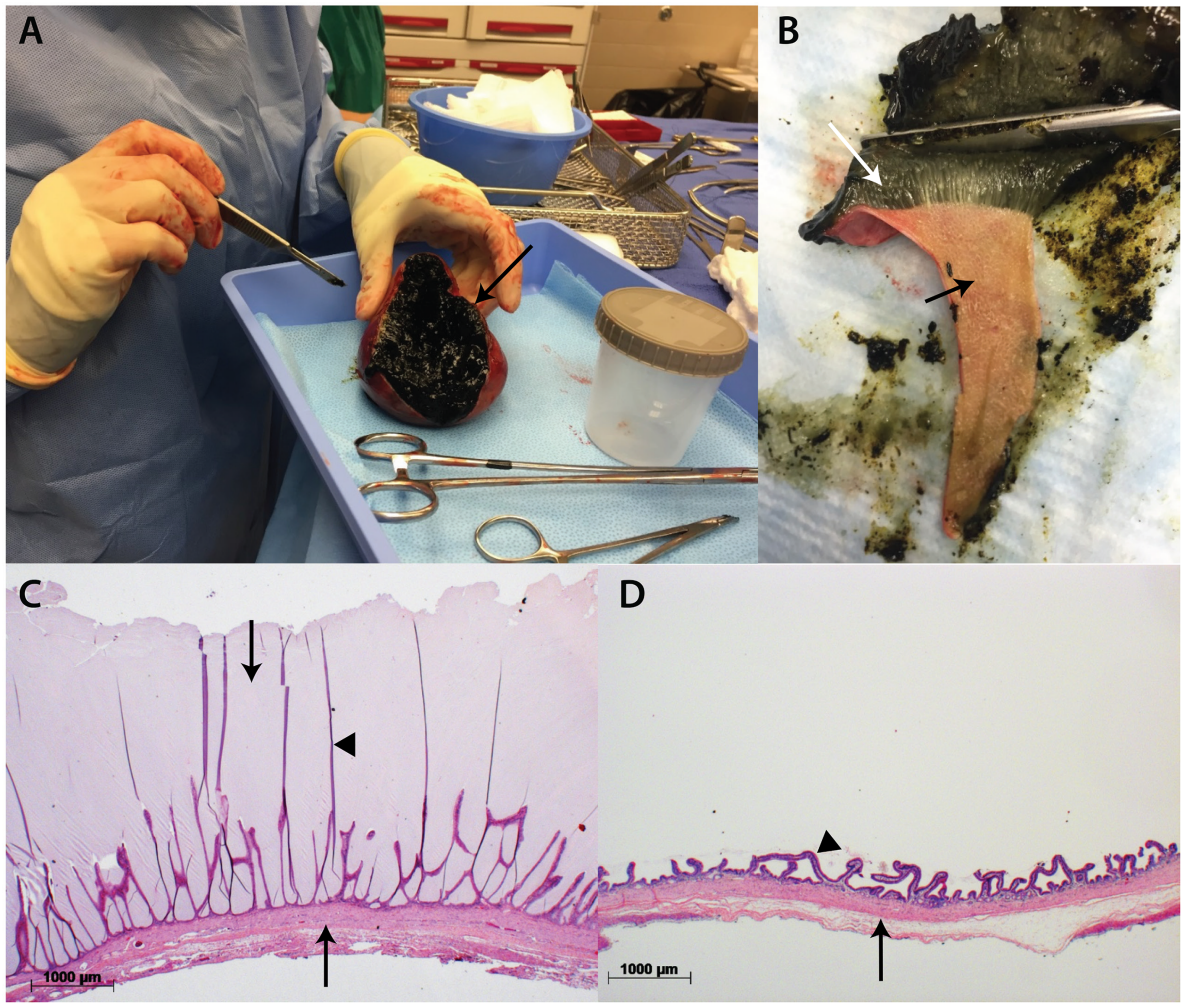
Gross and microscopic appearance of the internal contents of a gallbladder mucocele. Gross appearance of the internal contents of a mucocele immediately after surgical removal of the gallbladder from an affected dog (Panel A). In Panel A, the gallbladder has been incised from the neck to the apex along the margin indicated by the arrow resulting in exposure of the bile-stained, blackened mucus content. Panel B demonstrates firm adhesion of the gallbladder mucus (white arrow) as it is being “peeled” away from the lumen surface of the gallbladder mucosa (black arrow) using a scalpel. Panel C and D demonstrate a cross section of gallbladder mucosa (upward arrows) from a dog with mucocele formation and a control dog, respectively. In Panel C the gallbladder lumen (downward arrow) is effaced by solidified mucus into which extends thin papillary projections of the mucosal folds (closed arrowhead). In Panel D the lumen is devoid of mucus and normal mucosal folds can be visualized (closed arrowhead). Hematoxylin and eosin stain.

Grossly, all mucocele gallbladders were observed to be filled to distension by a semi-solid mass of gelatinous mucus. The mucus exhibited varying degrees of adhesion to the surface of the gallbladder mucosa (Fig 1A and 1B). Histologically, the gallbladder epithelium was in contact with uninterrupted layers of concentrically laminated mucus radiating from the surface of the epithelium into the lumen. The mucosal surface was thrown into tall, thin, simple papillary projections of epithelium supported by a thin fibrovascular stroma (n = 10)(Fig 1C and 1D). Additional findings were a few scattered lymphocytes and plasma cells within the submucosa (n = 5) and small numbers of pigment-filled macrophages present perivascularly (n = 2). Necrosis of the gallbladder epithelium consistent with ischemia and devitalization was reported in 3 dogs.

### Global mass spectrometry

A total of 350 named compounds were identified in serum of which 105 differed significantly (*p* ≤ 0.05; q < 0.10) between control dogs and those with mucocele formation. A total of 385 named compounds were identified in hepatic duct bile of which 121 differed significantly (*p* ≤ 0.05; q < 0.10) between control dogs and those with mucocele formation (Table 2). Median relative standard deviation of internal standards added to each sample prior to injection in the mass spectrometer was 6% for serum and 9% for bile samples. Median relative standard deviation of endogenous compounds in replicate samples of pooled serum and bile were 12% and 17%, respectively.

**Table 2:** Numbers of compounds in serum and hepatic duct bile that achieved statistical significance (*p* ≤ 0.05; q < 0.10), or approached significance (*p* = 0.05–0.10), when compared for differences between control dogs and those with mucocele formation using Welch’s two-sample *t*-test.

Named compounds	Mucocele Control	
	Serum	Bile
Total biochemicals (*p* ≤ 0.05)	105	121
Biochemicals (↑↓)	86 | 19	31 | 90
Total biochemicals (0.05 < *p*< 0.10)	26	49
Biochemicals (↑↓)	17 | 9	38 | 11

#### Amino acid metabolism

**Fig 2:**
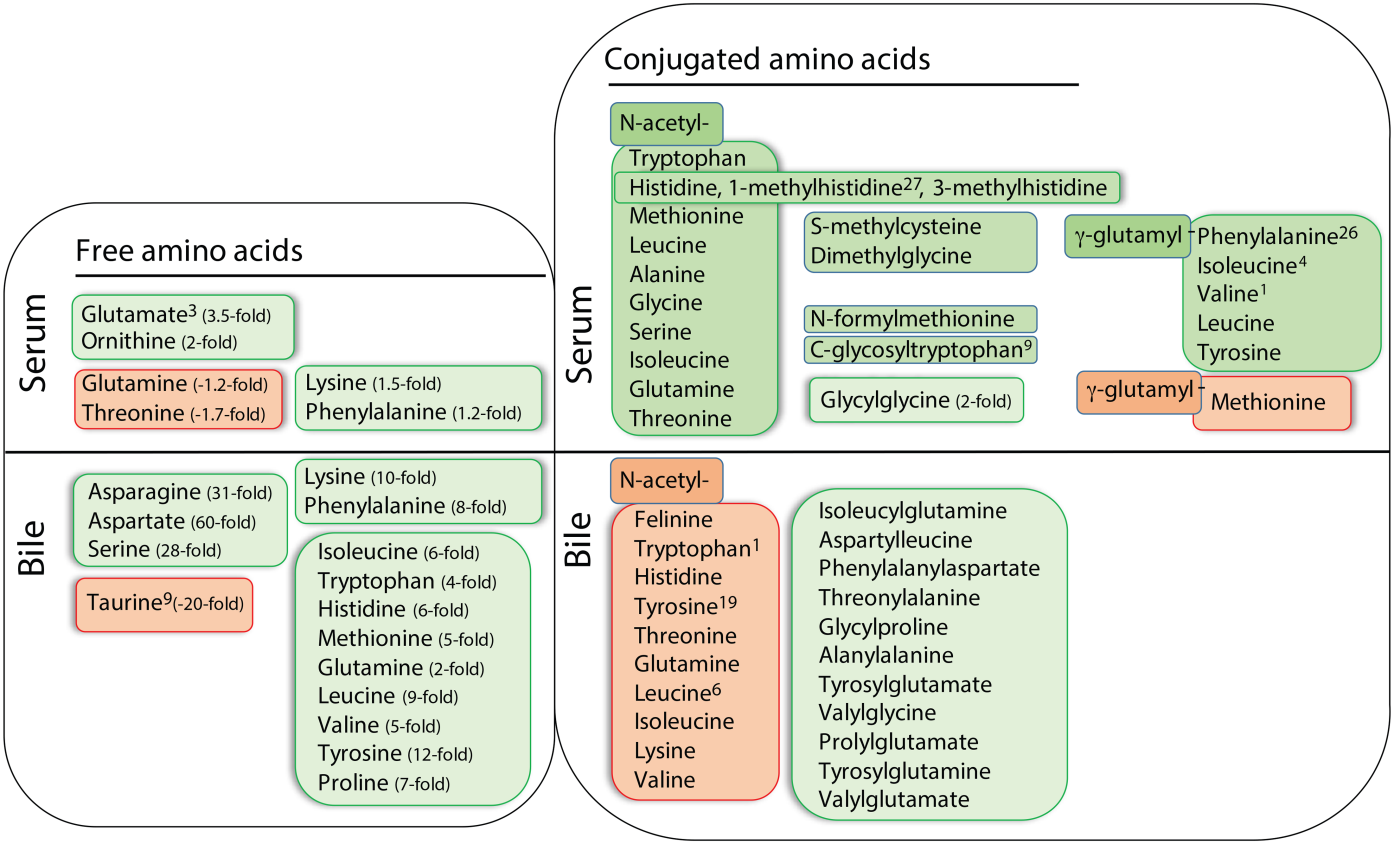
Free and conjugated amino acids identified to differ significantly in the serum or bile of dogs with gallbladder mucocele formation compared to control dogs. Free amino acids in serum and hepatic duct bile are shown on the left and conjugated amino acids in serum and hepatic duct bile are shown on the right. Compounds in green were increased and compounds shaded in red were decreased in dogs with gallbladder mucocele formation in comparison to control dogs. Superscript numbers, when present, indicate rank of the compound as identified by Random Forest analysis as able to distinguish between control dogs and dogs with gallbladder mucocele formation.

Remarkable disturbances in amino acid metabolism were observed in dogs with mucocele formation. Notably, numerous amino acids were identified in the hepatic duct bile from dogs with mucocele formation and in magnitudes ranging from 2 to 60-fold greater than observed in control dogs. Most of the amino acids excreted in the bile were significantly overrepresented in serum in the form of N-acetylated, γ-glutamylated, and in some cases methylated conjugates (S2 Table and Fig 2). Numerous dipeptides were also observed in hepatic duct bile of dogs with mucocele formation, while glycylglycine was the only dipeptide significantly increased in serum.

Individual amino acids of interest in bile of dogs with mucocele formation included the presence of a 60-fold increase in aspartate and a 30-fold increase in asparagine, the latter of which was detected in bile from 80% of dogs with mucocele formation and 0% of control dogs. Neither aspartate or asparagine was accompanied by serum retention of a conjugated derivative. Lysine and phenylalanine were significantly increased in both serum and bile of dogs with mucocele formation while glutamate, ornithine, and homocitrulline were increased only in serum. Specific factors suggesting that methionine may be limiting included increased amounts of methionine in bile and decreased amounts of γ-glutamyl methionine as compared to other γ-glutamyl amino acids in serum. Finally, dogs with mucocele formation had 20-fold less taurine in hepatic duct bile than observed for control dogs.

#### Glutathione and anti-oxidant activity

Serum of dogs with mucocele formation was characterized by significantly diminished amounts of oxidized glutathione and cysteine-glutathione disulfide. Notably, oxidized cysteinylglycine was 50-fold lower in bile of dogs with mucocele formation compared to control dogs. Abnormalities were also observed in the redox state of both vitamin C and E and were characterized by significant decreases in bile ascorbate and increases in serum alpha-tocopherol (S2 Table). No dogs in the study had a history of receiving either vitamin E or vitamin C supplements that could have accounted for these observations.

#### Carbohydrate and energy metabolism

In dogs with mucocele formation, abnormalities of glycolysis included greater than 2-fold increases in glycerate in both serum and bile and a 6-fold excretion of pyruvate into the bile. Three intermediates of the tricarboxylic acid cycle were significantly increased in the serum of dogs with mucocele formation including citrate, succinate, and fumarate. Citrate was also significantly increased in bile, however succinate and fumarate were significantly decreased in bile. Dogs with gallbladder mucocele formation had significant increases in serum and decreases in bile of a variety of diet and bacteria-derived sugar alcohols including erythritol, threitol, and xylonate. There were also increases in products of proteoglycan metabolism including serum N-acetylneuraminate (a sialic acid), glucuronate and erythronate. Several vitamins including pantothenate, riboflavin, and nicotinamide riboside were 3 to 10-fold lower in bile of dogs with mucocele formation compared to control dogs (S2 Table).

#### Nucleic acid metabolism

**Fig 3:**
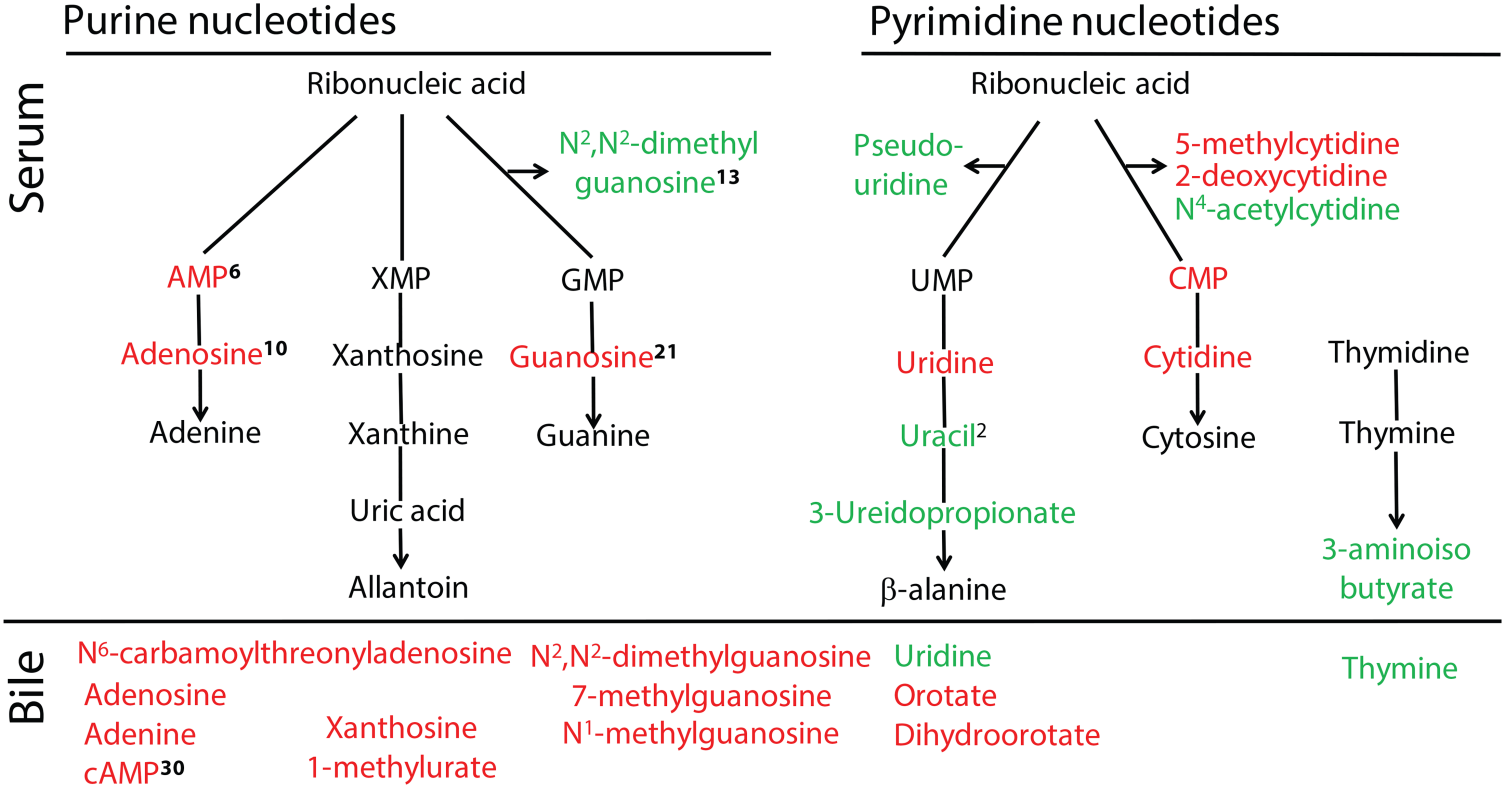
Significant differences in metabolites of ribonucleotide metabolism identified in the serum (top panel) and bile (bottom panel) of dogs with gallbladder mucocele formation compared to control dogs. Purine nucleotides are shown on the left and pyrimidine nucleotides are shown on the right. Compounds in green font were increased and compounds in red font were decreased in dogs with gallbladder mucocele formation in comparison to control dogs. Superscript numbers, when present, indicate rank of the compound as identified by Random Forest analysis as able to distinguish between control dogs and dogs with gallbladder mucocele formation.

Numerous intermediates of nucleotide metabolism differed significantly between control dogs and dogs with gallbladder mucocele formation (S2 Table and Fig 3). Significant decreases in both the purine and pyrimidine ribonucleosides was observed in serum. Notably, AMP and adenosine were 33-fold and 17-fold lower, respectively, in the serum of dogs with mucocele formation compared to control dogs. Similarly, cAMP, adenosine, and adenine were ≥ 2-fold lower in bile of dogs with mucocele formation. Significant differences were observed in the quantity of methylated ribonucleosides (e.g. N^2^,N^2^-dimethylguanosine, N^6^-carbomyl threonyladenosine, pseudouridine, N^1^-methylguanosine, and N^4^-acetylcytidine) and compounds needed for translation initiation (7-methylguanosine and N-formylmethionine) in the serum or bile of dogs with gallbladder mucocele formation compared to control dogs. Significant increases in uridine and thymine were observed in bile of dogs with mucocele formation.

#### Cholesterol and bile acid metabolism

**Fig 4:**
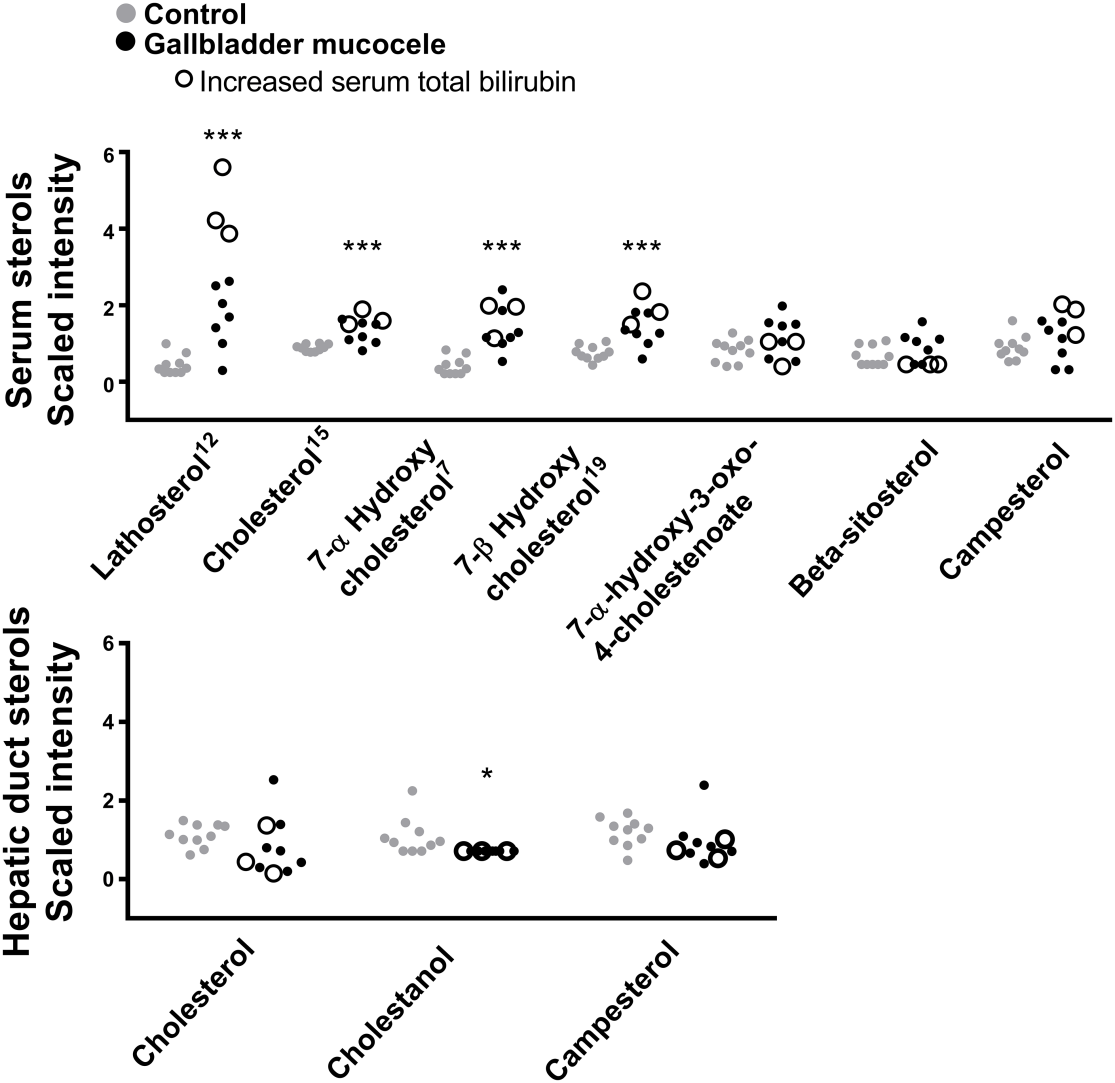
Compounds related to cholesterol metabolism identified in the serum (top panel) and hepatic duct bile (bottom panel) of dogs with gallbladder mucocele formation compared to control dogs. Data points represent individual dogs. Dogs with mucocele formation and a concurrent increase in serum total bilirubin are designated with open circles. Superscript numbers, when present, indicate rank of the compound as identified by Random Forest analysis as able to distinguish between control dogs and dogs with gallbladder mucocele formation. *p<0.05, ***p<0.001 Welch’s two sample t-test.

**Fig 5:**
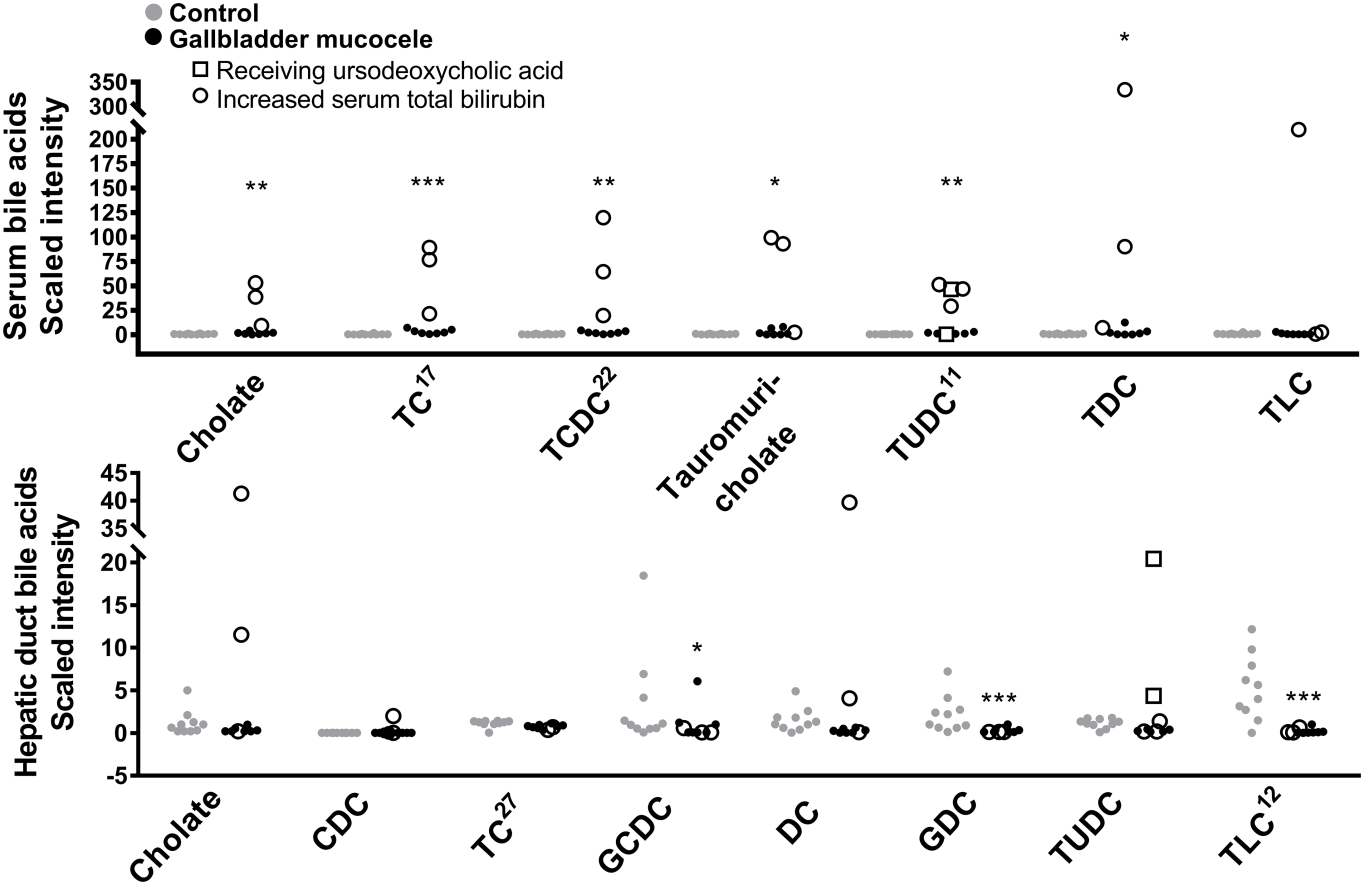
Compounds related to bile acid metabolism identified in the serum (top panel) and hepatic duct bile (bottom panel) of dogs with gallbladder mucocele formation compared to control dogs. Data points represent individual dogs. Dogs with mucocele formation and a concurrent increase in serum total bilirubin are designated with open circles. Superscript numbers, when present, indicate rank of the compound as identified by Random Forest analysis as able to distinguish between control dogs and dogs with gallbladder mucocele formation. *p<0.05, **p<0.01, ***p<0.001 Welch’s two sample t-test. TC, taurocholate; TCDC, taurochenodeoxycholate; TUDC, tauroursodeoxycholate; TDC, taurodeoxycholate; TLC, taurolithocholate; CDC, chenodeoxycholate; GCDC, glycochenodeoxycholate; DC, deoxycholate; GDC, glycodeoxycholate.

Dogs with gallbladder mucocele formation had significant increases in serum lathosterol, cholesterol, and 7-hydroxycholesterol. Increases in serum cholesterol could not be simply attributed to cholestasis as greater quantities were also observed in dogs with and without concurrent increases in serum total bilirubin or bile acids (Fig 4). In contrast, bile cholesterol was decreased compared to control dogs. Significant increases were observed in serum for nearly all bile acids that were detected (Fig 5), which included a 20-fold increase in the unconjugated bile acid cholate, ~40-fold increases in the taurine-conjugated bile acids taurocholate, taurochenodeoxycholate, tauro (alpha+beta) muricholate, and tauroursodeoxycholate, and a 67-fold increase in taurodeoxycholate. Notably, increases in serum bile acids could be specifically attributed to 3 individual dogs that had mild increases in serum total bilirubin or that were receiving ursodeoxycholic acid. A concomitant increase in bile acids in the bile of dogs with mucocele formation was not observed. Compared to control dogs, dogs with mucocele formation had a 25-fold decrease in taurolithocholate and a significantly lower quantity of the glycine-conjugated bile acids glycochenodeoxycholate and glycodeoxycholate in bile (S2 Table and Fig 5).

#### Lipid metabolism

Numerous abnormalities of lipid metabolism were observed in dogs with mucocele formation including significant increases in odd-chain, long-chain saturated, and polyunsaturated fatty acids (PUFA) in both serum and bile (S2 Table). Many of the PUFA were omega-3 fatty acids including the essential fatty acids linolenate, eicosapentaenoate, docosapentanoate, and docosahexaenoate. Increases in bile were also observed for the essential omega-6 fatty acids linoleate and arachidonate, but without concomitant increases in serum. Increases in the amount of free fatty acids in association with concurrent increases in lysophosphatidylcholines (lysoPC), glycerophosphorylcholine, glycerol-3-phosphate, glycerol, and choline, collectively support an increase in hydrolysis of phosphatidylcholine in dogs with mucocele formation. Phosphatidylcholine was not identified by global mass spectrometry in serum or bile from either group of dogs. Notably, 1-stearoylglycerophosphoserine, a specific lysolipid of phosphatidylserine, was observed to be 102.9 fold greater in the bile of dogs with mucocele formation compared to control dogs. Serum of dogs with mucocele formation had significant decreases in deoxycarnitine and 3-dehydrocarnitine, precursors for carnitine synthesis, and increases in acylcarnitines. A number of bioactive lipids were also observed to be significantly altered in serum or bile of dogs with mucocele formation including stearamide, N-acyl-taurine endocannabinoids, diacylglycerols, and stearoyl sphingomyelin.

#### Xenobiotics

Significant differences in the presence of several known xenobiotics including compounds related to benzoate metabolism, food components, products of hemoglobin metabolism, and exogenous drugs and chemicals were also identified in dogs with mucocele formation (S2 Table).

### Discriminating biomarkers of gallbladder mucocele formation

**Fig 6:**
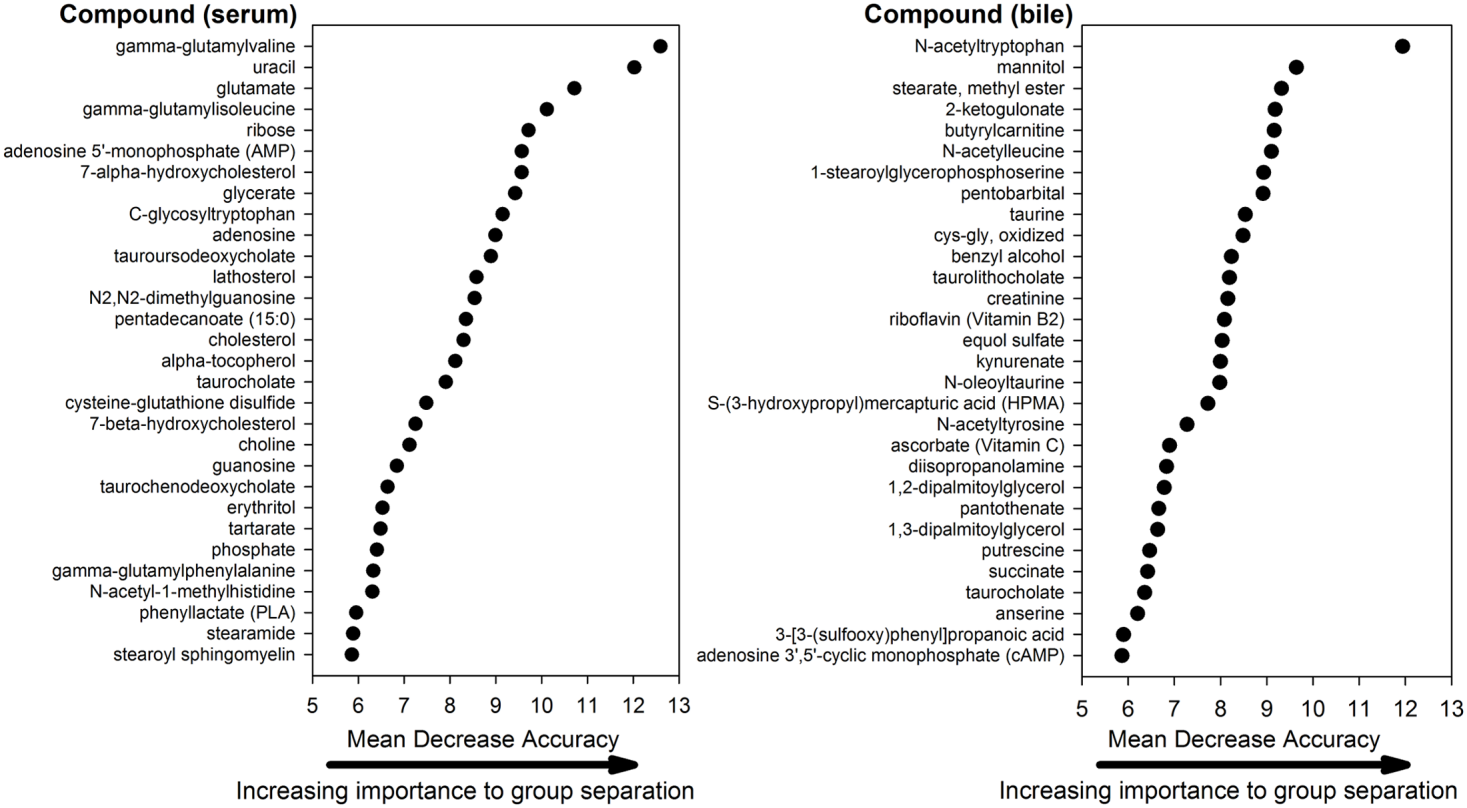
Top 30 metabolites in serum (left panel) and bile (right panel) in rank order of importance to distinguishing between control dogs and dogs with gallbladder mucocele formation. Data were generated by Random Forest analysis. Compounds contributing the most to the accuracy of disease classification were identified as those having the highest mean decrease accuracy (MDA) of the predicted classification when permuted within the dataset.

**Fig 7:**
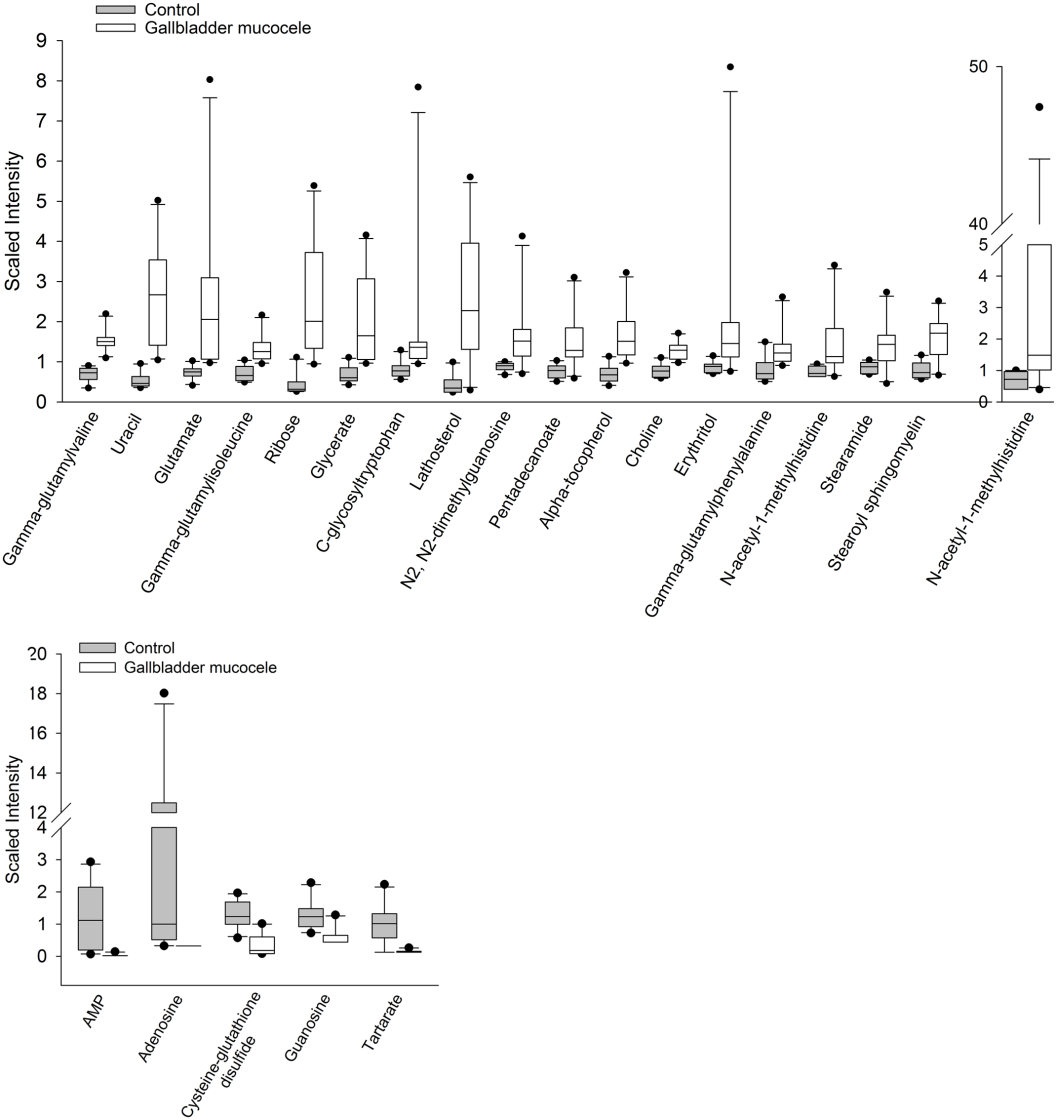
Selected serum metabolites with potential value as biomarkers of gallbladder mucocele formation. Each metabolite was identified by Random Forest analysis as important for classification of dogs into either control or gallbladder mucocele formation groups. Panel A demonstrates compounds observed to be significantly higher in the serum of dogs with mucocele formation. Panel B demonstrates compounds observed to be significantly lower in the serum of dogs with mucocele formation compared to control dogs. Data represent median, 25^th^ to 75^th^ percentile (box), 10^th^ to 90^th^ percentile (whiskers), and outlier data points.

Random forest analysis identified metabolites that were able to predict disease classification (control vs. mucocele formation) with an accuracy of 95%. Individual compounds in serum and bile that contributed the most to the accuracy of disease classification are shown in Fig 6. A subset of serum metabolites that were observed to be significantly altered in dogs with mucocele formation compared to control dogs were identified as potential biomarkers of disease (Table 3 and Fig 7). This subset was generated from the serum metabolites identified by random forest analysis followed by removal of compounds apt to be non-specifically increased in dogs with cholestasis (i.e. related to cholesterol or bile acid metabolism).

**Table 3:** Random Forest disease classification of the top 30 compounds identified in serum as contributing the greatest in distinguishing between dogs with mucocele formation and control dogs. Directionality of fold changes represent increases or decreases in the serum of dogs with mucocele formation compared to control dogs.

Rank Order	Serum Metabolite	Fold Change	P-value	Description
1	Gamma-glutamylvaline	2.25	6.34E-06	Flavor enhancer “kokumi” compound. Activates a calcium-sensing receptor (CaSR) that is also sensitive to glutathione.
2	Uracil	4.89	8.99E-07	Increased pyrimidine metabolism. Component of pantothenate and conenzyme A synthesis.
3	Glutamate	3.51	0.0005	Non-essential amino acid. Precursor for synthesis of GABA. Neurotransmitter. Substrate of transamination reactions. Positively associated with age in humans.
4	Gamma-glutamylisoleucine	1.88	2.17E-05	Gamma-glutamylated amino acid.
5	Ribose	5.95	1.22E-06	Pentose sugar. Essential component of nucleic acids, ATP, NAD and ADP-ribose.
6	Adenosine 5'-monophosphate (AMP)	-33.3	1.24E-05	Monomer of RNA. Index of low cellular energy. Activator of AMP-kinase.
7	7-alpha-hydroxycholesterol	3.58	1.22E-05	Product of cholesterol hydroxylation by CYP7A1 and precursor to synthesis of primary bile acids.
8	Glycerate	2.92	0.0002	Intermediate in conversion of serine to pyruvate.
9	C-Glycosyltryptophan	2.38	0.0084	Positively correlated with chronological age in humans.
10	Adenosine	-16.7	0.0063	Extracellular signaling molecule. Activates purinergic receptors to stimulate cAMP signaling.
11	Tauroursodeoxycholate	45.16	0.0031	Taurine conjugate of the secondary bile acid, ursodeoxycholic acid.
12	Lathosterol	5.81	0.0001	Precursor to synthesis of cholesterol.
13	N^2^,N^2^-dimethylguanosine	1.87	0.0057	Methylated ribonucleotide degradation product of tRNA metabolism.
14	Pentadecanoate (15:0)	1.97	0.0018	Odd-chain exogenous fatty acid of bacterial or milk fat origin.
15	Cholesterol	1.56	0.0005	Precursor for synthesis of cell membranes, steroid hormones, and bile acids.
16	Alpha-tocopherol	2.4	4.13E-05	Lipid soluble. Vitamin E.
17	Taurocholate	42.75	0.0004	Taurine-conjugated primary bile acid.
18	Cysteine-glutathione disulfide	-3.7	0.0003	Oxidation product of gluthathione.
19	7-Beta-hydroxycholesterol	1.97	0.0005	Bioactive lipid. Oxidation product of cholesterol.
20	Choline	1.63	3.62E-05	Methyl donor. Key component of phosphatidylcholine and acetylcholine.
21	Guanosine	-2.13	0.0002	Purine nucleotide.
22	Taurochenodeoxycholate	44.42	0.0019	Taurine-conjugated primary bile acid.
23	Erythritol	2.44	0.0117	Inedible carbohydrate, sugar alcohol, sugar substitute. Positively associated with age in humans.
24	Tartarate	-6.67	0.0004	Food additive and antioxidant. Synthesized from ascorbate in some plants.
25	Phosphate	1.55	0.0012	Inorganic ion and cellular buffer. After organification, is involved in substrate-level energy transfer and regulation of enzyme function.
26	Gamma-glutamylphenylalanine	1.63	0.0041	Gamma-glutamylated amino acid.
27	N-Acetyl-1-methylhistidine	11.72	0.013	Higher levels are associated with lower glomerular filtration rate in African Americans.
28	Phenyllactate (PLA)	1.93	0.0071	Metabolite of phenylalanine and tyrosine.
29	Stearamide	1.66	0.0189	Fatty acid amide; potential signaling molecule. Found in food packaging materials.
30	Stearoyl sphingomyelin	2.05	0.0004	Sphingolipid directly involved in intracellular vesicle trafficking due to specific recognition by p24 proteins and G-protein coupled receptors.

In addition to specifying the involvement of a given pathway in metabolic disruption, biologically active compounds identified in bile are of particular interest due to their potential for direct cellular effects on the gallbladder epithelium (Table 4). All but 2 of the most highly ranked compounds in bile were observed to be present in lower amounts in dogs with mucocele formation.

**Table 4:** Random Forest disease classification of the top 30 compounds identified in hepatic duct bile as contributing the greatest in distinguishing between dogs with mucocele formation and control dogs. Directionality of fold changes represent increases or decreases in the bile of dogs with mucocele formation compared to control dogs.

Rank Order	Bile Metabolite	Fold Change	P-value	Description
1	N-acetyltryptophan	-5.6	1.01E-05	N-acetylated amino acid. Neurokinin 1 (NK_1_) receptor antagonist.
2	Mannitol	-6.3	0.0002	Sugar alcohol.
3	Stearate, methyl ester	3.2	3.02E-05	Methyl ester fatty acid found in biodiesel.
4	2-Ketogulonate	-4.8	9.61E-05	Intermediate of ascorbic acid (vitamin C) metabolism in plants.
5	Butyrylcarnitine	-2.9	0.0001	Acylcarnitine. Reflective of excess carbon in mitochondria.
6	N-acetylleucine	-3.0	0.0002	N-acetylated amino acid.
7	1-Stearoyl glycerophosphoserine	103	0.0065	Lysophosphatidylserine that signals through G-protein coupled receptors to increase intracellular Ca^++^. Mast cell activator. Neutrophil signal to facilitate phagocytosis by macrophages.
8	Pentobarbital	-9.1	8.93E-05	Exogenous barbiturate.
9	Taurine	-20.0	7.70E-05	Sulfur amino acid used in conjugation of bile acids.
10	Cys-gly, oxidized	-50.0	2.49E-05	Thiol status indicator of oxidative stress.
11	Benzyl alcohol	-9.1	8.17E-05	Disinfecting agent.
12	Taurolithocholate	-25.0	0.0003	Taurine-conjugated secondary bile acid. Potent inhibitor of bile salt export pump (BSEP).
13	Creatinine	-7.7	0.0002	Break down product of creatine phosphate in muscle.
14	Riboflavin (Vitamin B2)	-5.0	0.0066	Coenzyme required for synthesis of flavin nucleotides FMN and FAD.
15	Equol sulfate	-33.3	0.0008	Soy-derived isoflavone with estrogenic properties. Interacts with BRCP/ABCG2 xenobiotic drug transporter.
16	Kynurenate	-4.2	0.003	Product of tryptophan metabolism. Antagonist of NMDA and α7 nicotinic acetylcholine receptors. Ligand for orphan G-protein-coupled receptor GPR35.
17	N-Oleoyltaurine	-3.6	0.0102	Endocannabinoid agonist of transient receptor potential (TRP) non-selective ion channels.
18	S-(3-hydroxypropyl) mercapturic acid (HPMA)	-4.2	2.00E-05	Metabolite from acrolein. Product of cigarette smoke. Used in the synthesis of methionine.
19	N-acetyltyrosine	-3.7	0.0073	N-acetylated amino acid.
20	Ascorbate (Vitamin C)	-16.7	0.0018	Vitamin C anti-oxidant.
21	Diisopropanolamine	-7.7	0.0002	Chemical emulsifying agent.
22	1,2-Dipalmitoylglycerol	-3.6	0.0002	Diacylglycerol. Signaling molecule derived from phospholipids. Activates protein kinase C.
23	Pantothenate	-3.2	0.0021	Vitamin B5. Precursor in the synthesis of coenzyme A (CoA).
24	1,3-Dipalmitoylglycerol	-3.6	0.0001	Diacylglycerol. Signaling molecule derived from phospholipids. Activates protein kinase C.
25	Putrescine	-8.3	0.0009	Polyamine.
26	Succinate	-33.3	0.0099	TCA cycle intermediate. Extracellular signaling molecule at G-protein coupled receptors.
27	Taurocholate	-1.6	0.4016	Taurine-conjugated primary bile acid.
28	Anserine	-2.1	0.0025	Product of histidine metabolism.
29	3-[3-(sulfooxy)phenyl] propanoic acid	-20.0	0.0012	Phenylsulfate.
30	Adenosine 3',5'-cyclic monophosphate (cAMP)	-5.6	0.0003	Intracellular second messenger signaling molecule produced by adenylyl cyclase.

### Compounds identified as relatively deficient in dogs with mucocele formation

**Fig 8:**
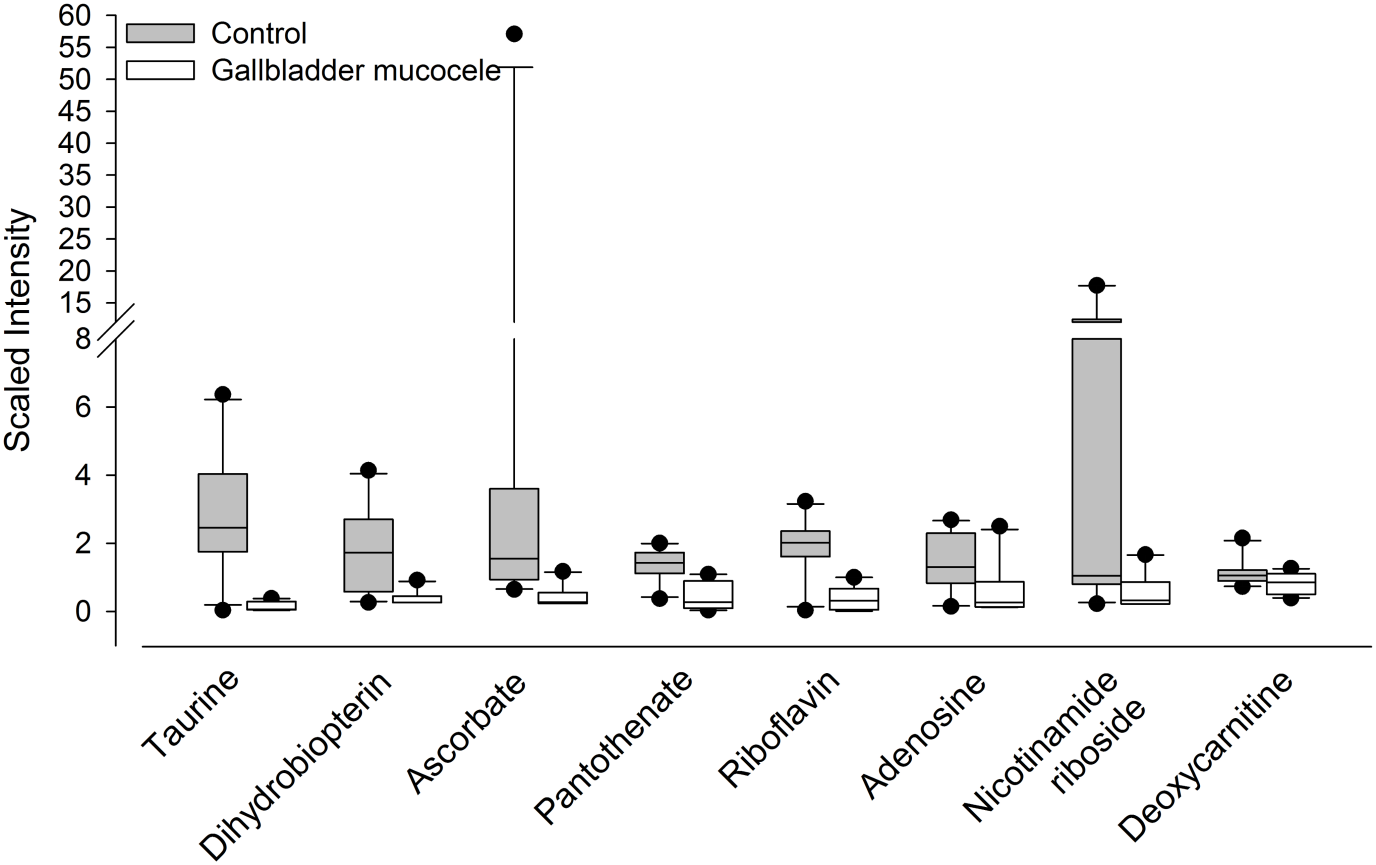
Cofactors, coenzymes, vitamins, and conditionally-essential compounds identified as significantly decreased in the serum or bile of dogs with mucocele formation compared to control dogs. Data represent median, 25^th^ to 75^th^ percentile (box), 10^th^ to 90^th^ percentile (whiskers), and outlier data points.

A number of compounds were identified as significantly less abundant in the serum or bile of dogs with mucocele formation compared to control dogs (Table 5 and Fig 8). In particular, there were relative decreases in the quantity of bile pantothenate and serum deoxycarnitine, which are related to the metabolism of parent compounds needed for the transport of energy substrates into mitochondria. Bile of dogs with mucocele formation had a 5 to 10-fold lower quantity of riboflavin and nicotinamide riboside, which are precursors for synthesis of the energy-transfer molecules FAD/FMN and NAD/NADP, respectively. Also in bile, were 17 to 20-fold decreases in the extracellular signaling molecule adenosine, the antioxidant ascorbate, and the bile acid conjugation substrate taurine.

**Table 5:** Compounds identified as relatively deficient in hepatic duct bile or serum from dogs with gallbladder mucocele formation relative to control dogs.

Metabolite	Location	Fold Change	P-value	Description
Taurine	Bile	-20.0	0.000077	Predominant amino acid used for conjugation of bile acids. Modulator of CYP7A1 activity.
Ascorbate	Bile	-16.7	0.0018	Vitamin C. Anti-oxidant. Biological regulator of CFTR-mediated Cl^-^ secretion.
Nicotinamide riboside	Bile	-10.0	0.0111	Precursor for synthesis of coenzymes NAD and NADP needed for energy-producing redox reactions.
Dihydrobiopterin	Bile	-5.0	0.0006	Precursor of tetrahydrobiopterin which is a required cofactor for conversion of aromatic amino acids to monoamine neurotransmitters and for synthesis of nitric oxide.
Riboflavin	Bile	-5.0	0.0066	Vitamin B2. Coenzyme required for synthesis of flavin nucleotides FMN and FAD.
Pantothenate	Bile	-3.2	0.0021	Vitamin B5. Precursor needed for synthesis of coenzyme A (CoA).
Adenosine	Serum	-16.7	0.0063	Extracellular signaling molecule. Activates purinergic receptors to stimulate CFTR-mediated Cl^-^ secretion.
Deoxycarnitine	Serum	-1.37	0.0495	Metabolite of carnitine. Carnitine is required for transport of fatty acids into mitochondria for β-oxidation.

## Discussion

### Metabolic insights into pathogenesis of gallbladder mucocele formation

**Fig 9:**
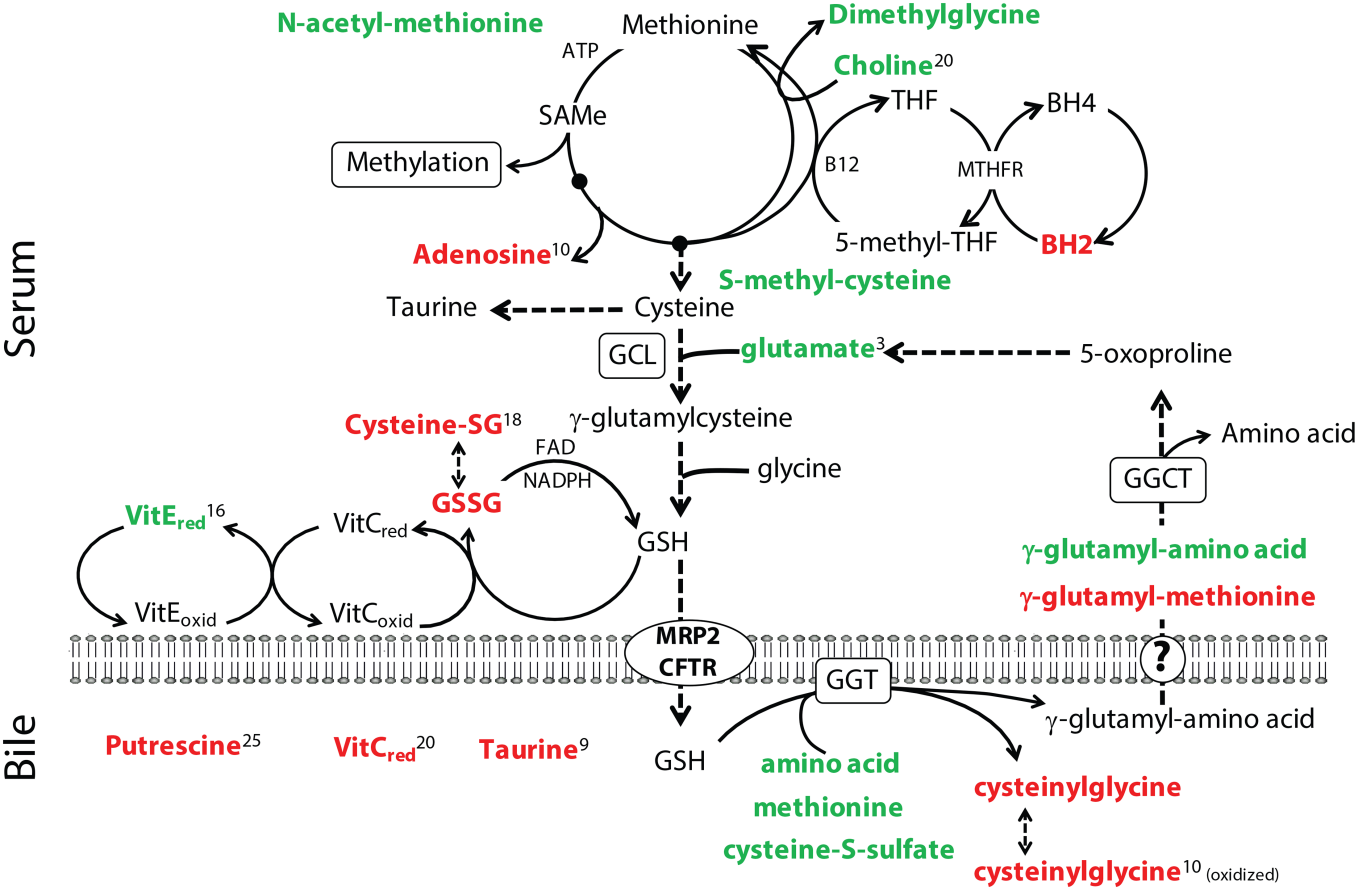
Hypothetical points of intersection of significantly altered compounds with one carbon metabolism, the methionine cycle, glutathione (GSH) synthesis and redox recycling in dogs with gallbladder mucocele formation. Top of Fig represents compounds identified in serum and bottom of Fig represents compounds identified in bile. Compounds in green font were increased and compounds in red font were decreased in dogs with gallbladder mucocele formation in comparison to control dogs. Superscript numbers, when present, indicate rank of the compound as identified by Random Forest analysis as able to distinguish between control dogs and dogs with gallbladder mucocele formation. THF, tetrahydrofolate; BH, hydrobiopterin; GGT, gamma-glutamyl transferase; GGCT, gamma-glutamyl cyclotransferase; GCL, glutamate cysteine ligase.

**Fig 10:**
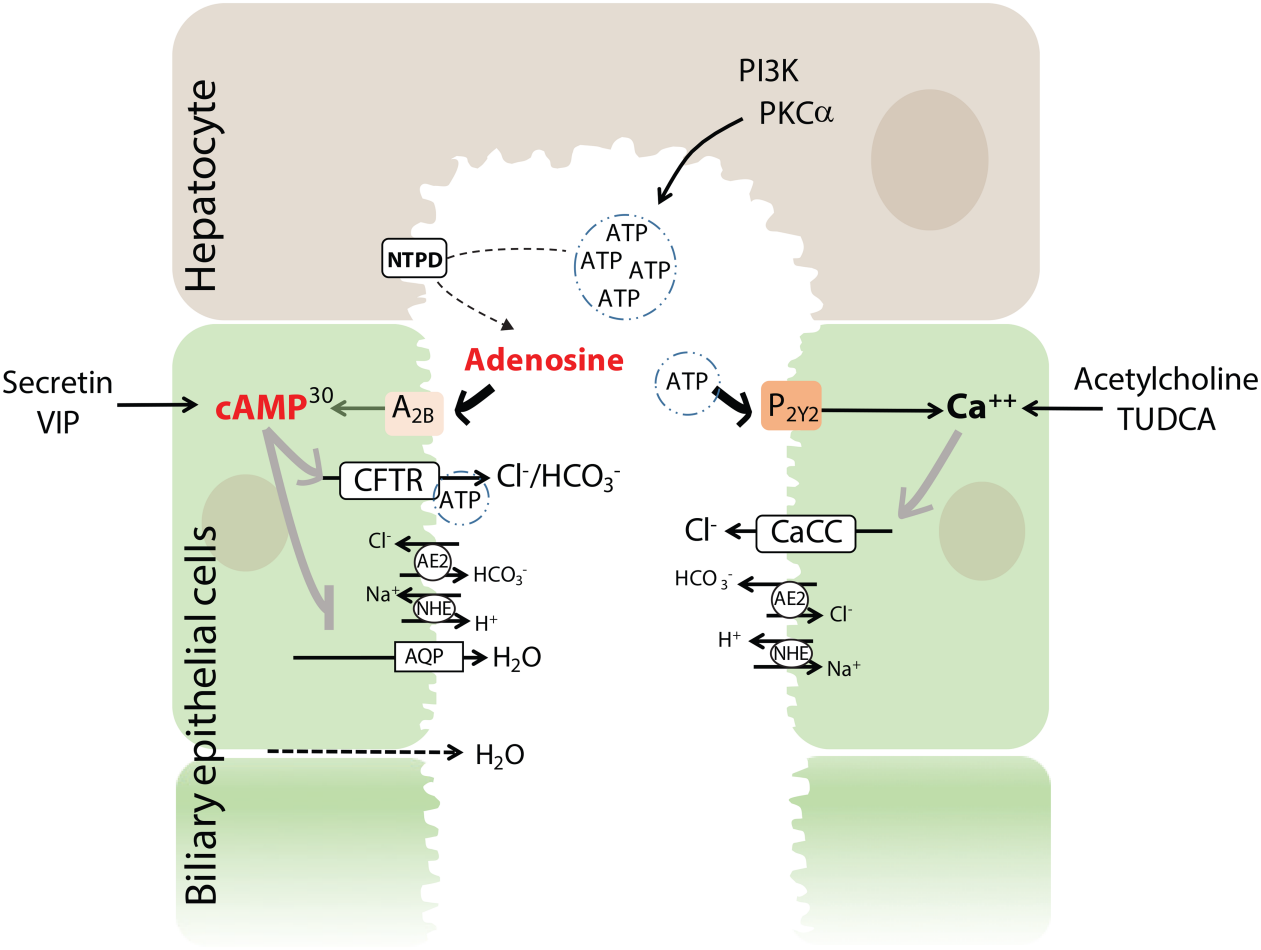
Role of ATP, adenosine, and cAMP in mediating chloride, bicarbonate, and water secretion by the biliary epithelium. Biliary canalicular secretion of ATP stimulates purinergic receptors (P_2Y2_) and calcium signaling which stimulates calcium-activated chloride secretory channels (CaCC). Hydrolysis of ATP by nucleoside triphosphate diphosphorohydrolase (NTDP) to adenosine stimulates nucleoside-selective receptors (A_2B_) and cAMP signaling which stimulates CFTR-mediated chloride secretion. Water is secondarily translocated into bile via transcellular (aquaporin channels; AQP) and paracellular pathways. Exogenous endocrine, paracrine and neurocrine stimuli of cAMP and calcium are also shown. Compounds in red font were decreased in dogs with gallbladder mucocele formation in comparison to control dogs. Superscript numbers, when present, indicate rank of the compound as identified by Random Forest analysis as able to distinguish between control dogs and dogs with gallbladder mucocele formation. Basolateral transport mechanisms are not illustrated. VIP, vasoactive intestinal polypeptide; TUDCA, tauroursodeoxycholic acid.

**Fig 11:**
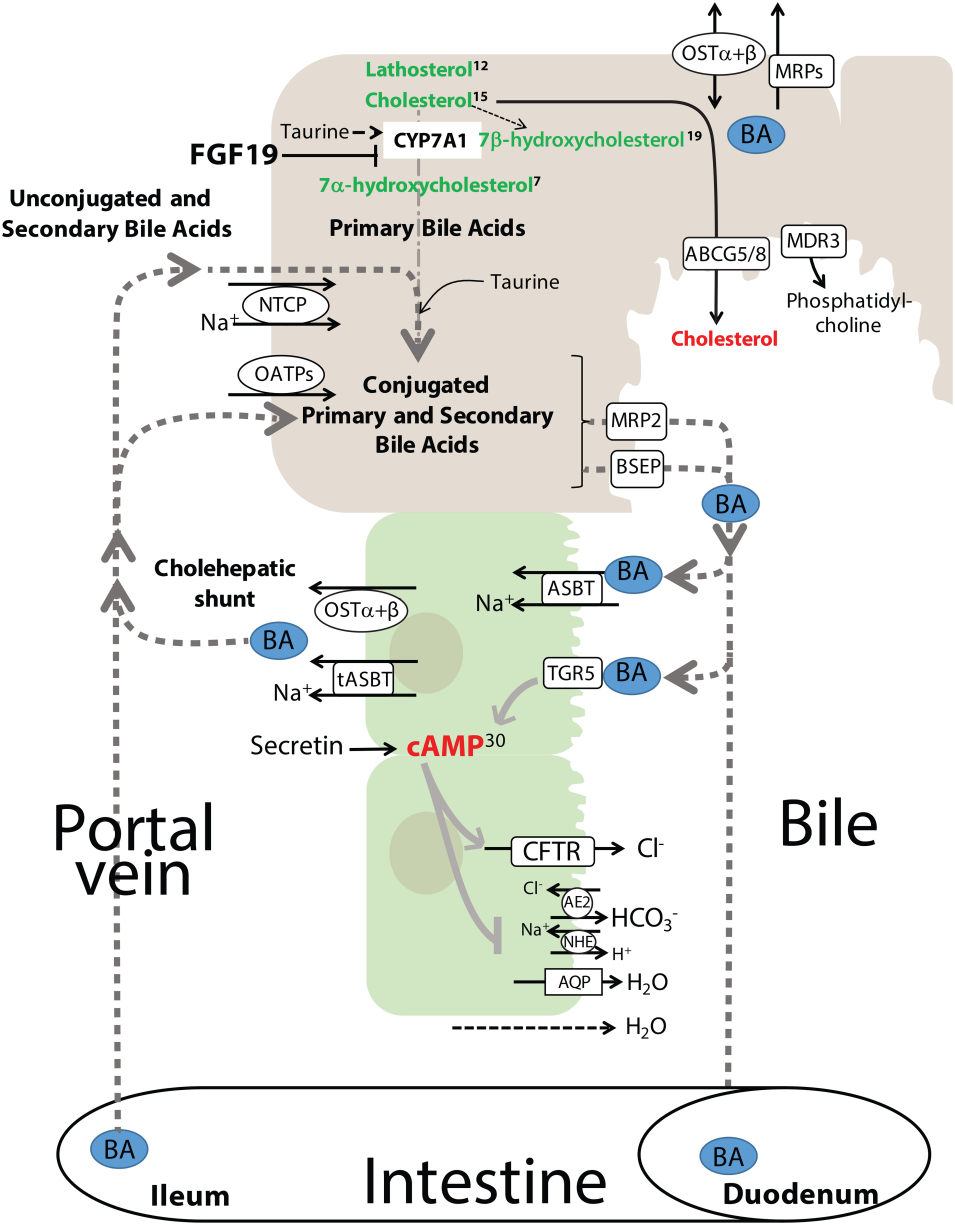
Significant alterations in cholesterol and bile acid metabolism in dogs with gallbladder mucocele formation. Fig depicts synthesis of cholesterol, conversion of cholesterol to primary bile acids, and taurine conjugation of primary and secondary bile acids taking place within an hepatocyte. Compounds identified as secreted into the bile are shown in the context of their interaction with the biliary epithelial cells. Bile acids interact with the biliary epithelium via transport (ASBT) and cell signaling receptors (TGR5). In particular, TGR5 stimulates increases in cAMP resulting in promotion of CFTR-mediated chloride secretion and inhibition of neutral NaCl absorption. Routes for both enterohepatic circulation and cholehepatic shunting of bile acids is shown. Compounds in green font were significantly increased while compounds in red font were significantly decreased in dogs with mucocele formation compared to control dogs. Superscript numbers, when present, indicate rank of the compound as identified by Random Forest analysis as able to distinguish between control dogs and dogs with gallbladder mucocele formation. BA, bile acids.

**Fig 12:**
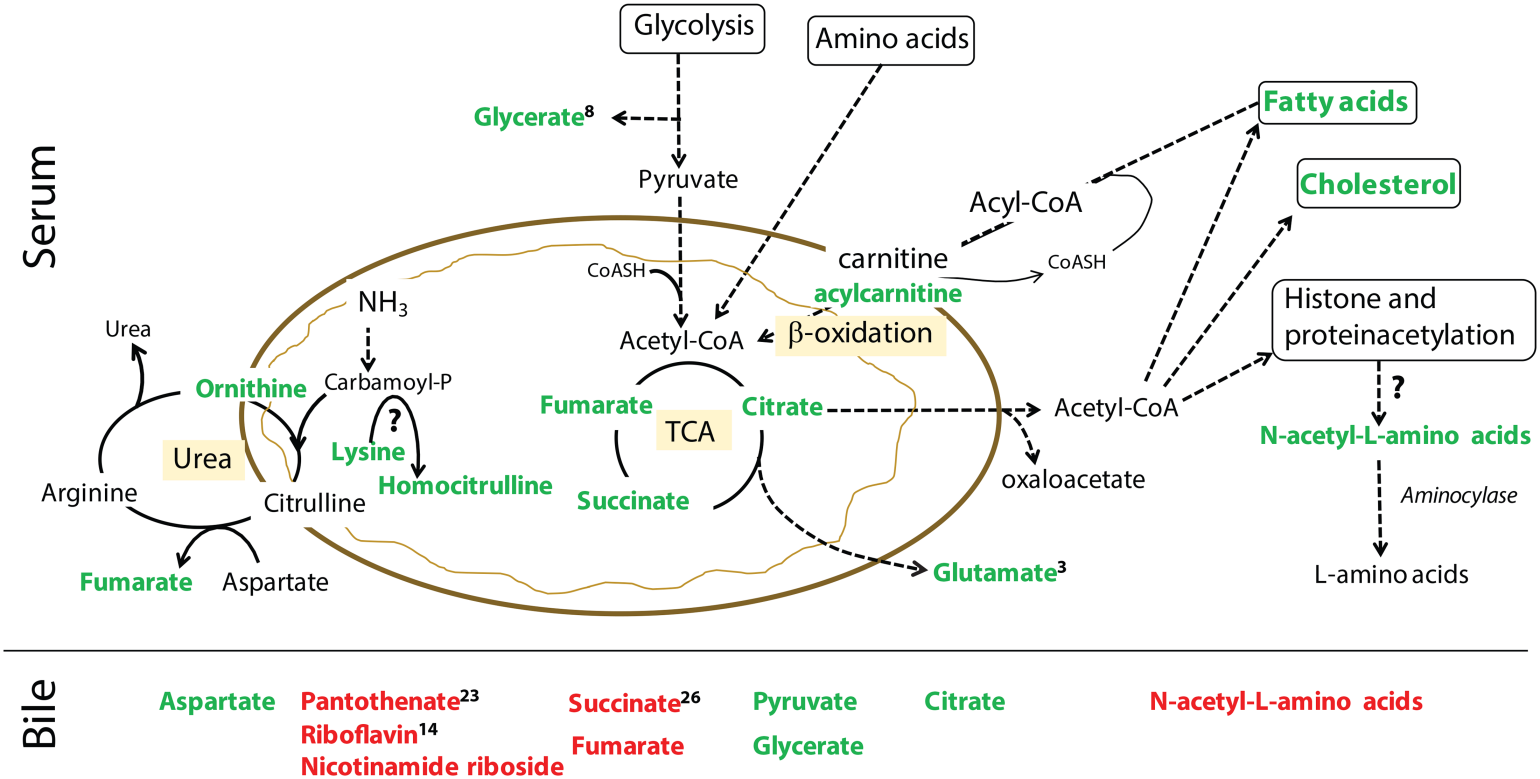
Hypothetical points of intersection of significantly altered compounds with carbon, nitrogen, and energy metabolism in dogs with gallbladder mucocele formation. Top of Fig represents compounds identified in serum and bottom of Fig represents compounds identified in bile. Compounds in green font were increased and compounds in red font were decreased in dogs with gallbladder mucocele formation in comparison to control dogs. Superscript numbers, when present, indicate rank of the compound as identified by Random Forest analysis as able to distinguish between control dogs and dogs with gallbladder mucocele formation. TCA, tricarboxylic acid cycle.

Results of this study document the presence of significant metabolic abnormalities in the serum and hepatic duct bile of dogs that were diagnosed with gallbladder mucocele formation. While this study understandably did not pinpoint the cause of mucocele formation in dogs, it provides unique insight into specific metabolic pathways that are disrupted. Moreover, concurrent examination of the serum and bile metabolome enables the construction of mechanism-based theories, as constructed in Figs 9–12, that can further localize the origins and potential impact of these abnormalities. In particular, individual compounds that are capable of exerting significant biological effects on function of the gallbladder epithelium were identified.

#### Abnormal amino acid and glutathione metabolism

Significant abnormalities were observed in the location, state of conjugation, and relative amounts of individual amino acids in dogs with mucocele formation compared to control dogs. Among these findings was the presence of increased amino acids in hepatic duct bile. The qualitative nature of our analysis does not provide absolute values for these amino acids. However, with the exception of glutamate, individual amino acids are reportedly present in low to undetectable concentrations in normal dog bile (generally < 100 μM)[30]. Amino acids are considered to be actively excreted when their bile concentrations are greater than observed in plasma[30, 31]. In dogs with mucocele formation, concurrent increases of aspartate, asparagine, lysine and phenylalanine in serum and bile, suggests that these amino acids may be produced in excess and actively eliminated in bile. The reason for the remaining amino acids to be significantly increased in bile is unclear. Dog gallbladder mucosa is capable of reabsorbing amino acids by a sodium-dependent transport mechanism. However, when the common bile duct is ligated, amino acid uptake capacity is greatly reduced[32]. Accordingly, it is possible that stasis of bile, depletion of sodium content, or abnormal function of the gallbladder epithelium may contribute to loss of amino acids into the bile of dogs with mucocele formation. Alternatively, there may be an increase in proteolysis within the bile of dogs with mucocele formation.

Increases in serum γ-glutamyl amino acids in dogs with mucocele formation suggest an involvement of γ-glutamyltransferase (GGT) in possible salvage of the free amino acids from bile (Fig 9). Alternatively, this could reflect an increased activity of glutamate-cysteine ligase[33]. Glutathione is a major secretory product of bile where it is extensively degraded within the biliary tree by GGT to facilitate reabsorption of its component amino acids (glutamate, cysteine, and glycine). The reaction involves transfer of the glutamate residue of glutathione to a free amino acid resulting in a γ-glutamylated amino acid and cysteinylglycine. Indirect evidence suggests that glutamate is reabsorbed from bile as a γ-glutamyl amino acid conjugate[30]. As such, GGT is speculated to additionally function as a mediator of amino acid transport[34]. Accordingly, increases in γ-glutamyl amino acid conjugates in serum of dogs with mucocele formation could reflect GGT-mediated salvage of amino acids from the bile.

An alternate explanation for increased free amino acids in bile could be a deficit of glutathione in bile, which is supported by a significantly lower quantity of cysteinylglycine in bile of dogs with mucocele formation (Fig 9). Glutathione is excreted in bile by multi-drug associated resistance protein 2 (MRP2; ABCC2) and the cystic fibrosis transmembrane regulator protein (CFTR)[35] and therefore its transport is susceptible to inhibition by xenobiotics or altered CFTR function[36]. Secretion of glutathione provides the primary osmotic force for bile salt-independent movement of water into the biliary canaliculi and is essential to detoxification of drugs, heavy metals, and oxidative compounds in bile[37]. Accordingly, a deficiency of glutathione in bile could directly contribute to decreased canalicular fluid secretion and impaired availability of glutathione to reduce the highly disulfide cross-linked mucus identified in the gallbladder of dogs with mucocele formation[22]. Based on these observations, a targeted quantification of biliary glutathione concentrations in dogs with mucocele formation is warranted. Documentation of biliary glutathione deficiency would provide a sound rationale for treating affected dogs with drugs such as S-adenosyl-methionine and would merit closer investigation of MRP2 and CFTR inhibition as potential culprits of mucocele formation.

The presence of significantly lower amounts of oxidized glutathione and cysteine-glutathione disulfide in the serum of dogs with mucocele formation, do not support an increase in systemic oxidative stress. This is similar to observations in people with cystic fibrosis where GSH levels in lung secretions are low, while levels in the lung tissue are unaffected[38]. However, significantly lower levels of L-ascorbate (vitamin C) in the bile of dogs with mucocele formation and lower L-ascorbate levels in serum suggests that quantitative analysis of this anti-oxidant vitamin is warranted (Fig 9). This is noteworthy as vitamin C has been identified as a biological regulator of CFTR-mediated Cl^-^ secretion in epithelia. Deficiency of vitamin C in people with cystic fibrosis has been proposed to further diminish airway hydration[39]. This observation suggests that supplementation of vitamin C might be beneficial in promoting mucus hydration in dogs with mucocele formation.

A noteworthy finding in dogs with mucocele formation was a significant increase in serum N-acetyl amino acids. At the time of synthesis, up to 90% of cellular proteins can undergo acetylation of the N-terminal amino acid. The reaction is catalyzed by N-acetyltransferase enzymes using acetyl-coenzyme A as the acetyl group donor[40]. The N-acetylated amino acids become liberated when the acetylated proteins are degraded[41]. Therefore, an increase in protein catabolism is likely responsible for serum elevations in N-acetyl amino acids in dogs with gallbladder mucocele formation. Importantly, N-acetyl amino acids cannot be directly re-used for new protein synthesis. Therefore, it is interesting that dogs with mucocele formation had significantly lower quantities of N-acetyl amino acids in bile compared to control dogs, suggesting an active effort to retain these amino acids. The kidneys are able to salvage N-acetyl amino acids from the renal tubular filtrate through the actions of aminoacylase-1 and return them as free amino acids to the plasma for re-use[42, 43]. Therefore, examination of urine of dogs with mucocele formation for the presence of N-acetyl amino acids may shed light on whether they are being produced in excess and excreted versus reclaimed by the kidneys, and perhaps from the bile, as free amino acids. Reclamation of these N-acetyl amino acids could represent a compensatory response to the loss of free amino acids into bile in dogs with mucocele formation.

#### Abnormal RNA and adenosine nucleotide metabolism

Dogs with gallbladder mucocele formation appear to have a decrease in substrates and increase in degradation products of RNA metabolism. Lower amounts of all 4 of the major ribonucleosides in the serum of dogs with gallbladder mucocele formation suggests that substrates needed for RNA synthesis may be limited. A decrease in 7-methylguanosine in bile suggests abnormal regulation of cap-dependent protein synthesis, as this compound serves as the 5’ cap that is needed for translation of most cellular mRNAs[44]. Other methylated ribonucleosides that were altered in dogs with mucocele formation are uniquely found in tRNA[45]. Since methylation occurs only after synthesis of the tRNA, individual methylated ribonucleosides represent end products of tRNA catabolism. Finally, increases in N-formylmethionine, which serves as the initiation amino acid for translation of mitochondrial protein[46] suggests a specific change in mitochondrial protein metabolism.

A possible culprit of abnormal protein metabolism in dogs with mucocele formation could be a disorder of methylation. Methylation is required for the correct function and stabilization of tRNA[47]. Abnormal methylation of tRNA leads to errors in protein translation and increased protein degradation. Central to methylation reactions is normal function of the methionine cycle (Fig 9). Altered methionine cycle function could also explain decreased synthesis of adenosine as well as abnormalities in the metabolism of taurine, glutathione, putrescine, and carnitine that were observed in dogs with mucocele formation. Moreover, methionine plays a specific role as the initiating amino acid in mRNA translation. These collective observations warrant a closer look at methionine homeostasis in dogs with gallbladder mucocele formation.

In addition to serving as a building block for nucleic acids, adenosine nucleotides intersect at numerous sites of metabolism including as a translocator of cellular energy in the form of ATP, as the second messenger cAMP, as a central regulator of metabolism in the form of AMP, precursor for the synthesis of NAD+, and in control of ATP-binding proteins and enzymes. Particularly noteworthy in this study was the significantly lower amount of adenosine and cAMP in the bile of dogs with mucocele formation. ATP is normally secreted into bile and hydrolyzed by hepatic canalicular ecto-ATPases[48] to form adenosine. Both ATP and adenosine serve as important extracellular signaling molecules that promote bile hydration by stimulating chloride, bicarbonate, and water secretion by the biliary epithelial cells. ATP binds to purinergic (P2Y) receptors to stimulate calcium-activated chloride channels (CaCC)[49] while adenosine binds to nucleoside-selective (A2B) receptors to stimulate cAMP-activated CFTR channels[50] (Fig 10). In the lung, surface liquid homeostasis is primarily maintained by continuous generation of adenosine that provides sustained input to the A2B receptor and stimulation of CFTR activity. Accordingly, a relative decrease in the amount of adenosine and cAMP in the bile of dogs with mucocele formation could portend impaired CFTR-mediated fluid secretion leading to mucus dehydration akin to that seen in people with cystic fibrosis. Alternatively, lower amounts of cAMP in dogs with mucocele formation could reflect decreased stimulation of the gallbladder epithelium by exogenous (secretin or vasoactive intestinal polypeptide (VIP)) or endogenous (bile acids) mediators that activate CFTR via cAMP[1]. Given the enormity of impact of these signaling pathways and ion transport mechanisms on maintenance of bile liquidity[1], electrophysiological studies of the gallbladder epithelium of dogs with mucocele formation are warranted.

#### Abnormal bile acid and cholesterol metabolism

The overall composition of serum bile acids in dogs with and without mucocele formation was similar to that reported in normal dogs in consisting of >99% taurine-conjugated primary and secondary bile acids[51–53]. With exception to dogs with cholestasis, dogs with mucocele formation had a similar quantity of bile acids in serum, but a lower quantity of some bile acids in hepatic duct bile compared to control dogs. Others have similarly shown decreased concentrations of bile acids in mucus collected from the gallbladder of dogs with mucocele formation[54]. Lower quantities of bile acids in hepatic duct bile of dogs with mucocele formation may reflect cholehepatic shunting of bile acids from the duct lumen back to the liver in an effort to augment bile salt dependent fluid secretion and prevent long exposures of the biliary epithelium to bile acids[55]. It has been theorized that exposure of the gallbladder epithelium to an altered bile acid composition may be a contributing factor to gallbladder mucocele formation in dogs[52]. In particular, hydrophobic bile acids stimulate cAMP-independent mucin secretion by dog gallbladder epithelial cells *in vitro*[56]. Importantly, no relative increases in hydrophobic or unconjugated bile acids (i.e. chenodeoxycholic or deoxycholic) were observed in the bile of dogs with mucocele formation in this study, which suggests consideration of alternate mechanisms for mucus hypersecretion.

After export from the hepatocytes, bile acids also play an important role in regulating ductular secretion by the biliary epithelial cells. In particular, ductular bile acids activate the G-protein coupled receptor TGR5 which transmits signals via the cAMP-signaling pathway[57]. Specific bile acids such as taurocholic and taurolithocholic acid stimulate ductal secretion by increasing expression of secretin receptors, cAMP levels, and Cl^−^/HCO_3_^−^ anion exchanger 2 (AE2) activity[58, 59]. Bicarbonate (HCO_3_^-^) then creates an osmotic gradient for aquaporin 1 to promote the efflux of water, leading to increased hydrocholeresis[60]. The resulting bicarbonate-rich secretion is responsible for creation of a protective “biliary bicarbonate umbrella”[61]. In comparing the bile acid composition of hepatic duct bile from dogs with mucocele formation to that of control dogs, random forest analysis identified decreases in both taurocholate and taurolithocholate as distinguishing features. Accordingly, a relative deficiency of these bile acids could conceivably impair secretin-mediated ductular fluid secretion and alkalinization. This possibility is indirectly supported by significant decreases in cAMP in bile of dogs with gallbladder mucocele formation. Based on this observation it is worthwhile to further investigate dogs with mucocele formation for the possibility of impaired secretin-mediated hydrocholeresis and/or failure to appropriately alkalinize bile. In cystic fibrosis, loss of CFTR-mediated HCO_3_^–^ secretion leads to airway dehydration and an abnormally low surface liquid pH that increases mucus viscosity and failure of mucus to break free following exocytosis by the epithelium[24, 62].

Dogs with gallbladder mucocele formation are commonly prescribed ursodeoxycholic acid (UDC) in an effort to stimulate choleresis and displace the more hydrophobic endogenous bile acids from the enterohepatic recirculating pool[63]. After absorption by the intestinal tract, UDC becomes taurine-conjugated in the liver to form tauroursodeoxycholic acid (TUDC). In this study, TUDC was increased only in the serum of dogs with cholestasis or those receiving exogenous UDC. Both dogs receiving UDC also had increased quantities of TUDC in hepatic duct bile suggesting that UDC administration to dogs with mucocele formation is effective in increasing bile TUDC content.

Hypercholesterolemia is common in dogs with mucocele formation[18] and was documented by serum biochemistry to be above reference range limits in 40% of the dogs with mucocele formation in this study. Using mass spectrometry, cholesterol was confirmed to be significantly greater in serum of dogs with mucocele formation compared to control dogs but was not concurrently increased in bile. The major route for elimination of cholesterol is directly into bile using the canalicular bidirectional sterol transporter ABCG5/8[64, 65] or by conversion to bile acids (Fig 11). Inhibition of transport of cholesterol into bile or enhanced reabsorption by the biliary epithelium could explain higher serum cholesterol and comparatively low bile cholesterol. However, a significant increase in lathosterol, a cholesterol precursor, suggests a primary increase in cholesterol synthesis. Moreover, significant increases in 7α-hydroxycholesterol support CYP7A1-associated diversion of cholesterol to bile acid synthesis. Continued CYP7A1 activity despite elevated serum bile acids in dogs with cholestasis is counterproductive and suggests a failure of feedback inhibition. Feedback inhibition is initiated by bile salt activation of farnesoid X receptor (FXR) signaling in the small intestine resulting in release of fibroblast growth factor 19 (FGF19). FGF19 returns to the liver through the portal circulation to repress CYP7A1 activity. It is possible that lower amounts of bile acids in the intestinal tract of dogs with mucocele formation lead to diminished secretion of FGF19 and consequently unchecked synthesis of primary bile acids from cholesterol. An increased demand for taurine for synthesis of these bile acids might explain the 20-fold lower amounts of taurine observed in the bile of dogs with gallbladder mucocele formation.

#### Abnormal lipid and energy metabolism

Significant abnormalities in lipid metabolism lend additional insight into the origins of hyperlipidemia in dogs with mucocele formation. Phosphatidylcholine (PC) was not identified in serum or hepatic duct bile from either group of dogs and therefore it remains unclear if quantities of PC are abnormal in dogs with mucocele formation. This assessment is worthy of targeted analysis due to the importance of PC in bile. In dogs, PC constitutes 94.5% of total biliary phospholipid[66] and functions to form mixed micelles with bile acids and cholesterol to promote their solubility and excretion. Hydrolysis of PC to lysoPC (normally absent in bile[66]), is described in people with a variety of hepatobiliary disorders and is attributed to increased activity of secretory low–molecular weight phospholipase A_2_ (sPLA_2_) in bile[67]. As a key enzyme in release of arachidonic acid (AA), increased bile sPLA_2_ could also explain elevations of AA observed in the bile of dogs with mucocele formation. Importantly, increases in lysoPC, AA, and AA-derived eicosanoids (e.g. PGE_2_) in bile are each associated with hypersecretion of biliary mucin in naturally-occurring and experimental models of gallbladder disease[68–70] [71]. Increases in intracellular AA can also serve as a potent inhibitor of CFTR[72, 73].

Increased amounts of odd-chain fatty acids in the serum of dogs with mucocele formation indicates that some of these lipids are of exogenous origin as animals assemble >99% of their fatty acids in two-carbon units[74]. Odd-chain saturated fatty acids are thought to originate from ingestion of dairy (milk) fat which contains odd-chain fatty acids produced by rumen microbes[75]. Increases in odd-chain fatty acids are also observed secondary to disorders that inhibit mitochondrial propionyl-CoA metabolism[76]. Along these lines, mitochondrial dysfunction was suggested by increases in serum acylcarnitines (e.g. butyrylcarnitine) which largely reflect removal of excess carbon from the mitochondria due to an accumulation of acyl-CoA’s. Moreover, decreased amounts of synthetic precursors such as deoxycarnitine may portend taxation of ongoing carnitine synthesis in dogs with mucocele formation.

In several respects dogs with gallbladder mucocele formation demonstrate disturbances in cellular energy and redox balance similar to those described in people with metabolic syndrome. Remarkably, a 33-fold decrease in serum adenosine 5’-monophosphate (AMP) suggests that dogs with mucocele formation are in a state of excess metabolic energy. In fact, lower serum AMP was identified by random forest analysis as one of the most distinguishing serum abnormalities identified in dogs with mucocele formation. Significant increases in citric acid cycle intermediates in dogs with mucocele formation suggest a surplus of carbon flow into the cycle. Under such conditions acetyl-CoA is removed from the mitochondria in the form of citrate and its carbon used for storage as sterols, fatty acids, and for promotion of acetylation reactions[77, 78] (Fig 12). Significantly lower amounts of pantothenate, riboflavin, and nicotinamide riboside in the bile of dogs with mucocele formation suggest an increased need for use of these precursors for synthesis of coenzyme-A and the energy transporting nucleotides FAD, FMN, and NAD+. As these coenzymes play critical roles in the transfer of metabolic energy, supplementation of these vitamins in dogs with mucocele formation should be considered.

### Potential biomarkers of gallbladder mucocele formation

A number of compounds in serum and hepatic duct bile were individually identified for their value in distinguishing between control dogs and those that formed a gallbladder mucocele. Bile of dogs with mucocele formation was noteworthy for lower amounts of many compounds with known biological effects on biliary epithelium. This would suggest that, if relevant, their influence on the gallbladder epithelium would be attributed to their relative deficiency. The compound with the greatest fold increase in bile of dogs with mucocele formation was 1-stearoylglycerophosphoserine (lysoPS). LysoPS is a signaling lipid that is generated by phospholipase A_2_-mediated hydrolysis of phosphatidylserine. LysoPS can signal through several orphan G-protein coupled P2Y purinergic receptors that increase intracellular calcium and inhibit intracellular cAMP[79]. While most research to-date suggests restriction of lysoPS receptor expression to immune cells, the effect of lysoPS on gallbladder epithelial cells is unknown. Given the 103-fold increase in lysoPS in bile of dogs with mucocele formation, and recognized role of calcium signaling on mucus secretion, the biological effects of this compound on gallbladder epithelial secretion is worth exploring.

In contrast to bile, compounds identified in serum are of added interest due to their potential value as measurable biomarkers. Mucocele formation is slowly progressive and early clinical signs can overlap with more common and self-limiting gastrointestinal diseases. By the time clinical signs are overt, the gallbladder is frequently ruptured or bile flow is obstructed and surgery becomes the only treatment option. At present, ante mortem diagnosis of gallbladder mucocele formation is entirely reliant on the use of abdominal ultrasound. Despite a heightened awareness of age and breed predispositions, ultrasonography is too expensive and impractical for routine screening of dogs for mucocele formation. Therefore, discovery of serum biomarkers of mucocele formation would enable more frequent screening of predisposed dogs, earlier diagnosis for improved surgical prognosis, and provide an extended opportunity for medical intervention. In this study, potential serum biomarkers were represented by a number of biologically active compounds, compounds reflective of increased cholesterol and bile acid synthesis, substances of exogenous origin, and metabolites suggestive of carbon and energy excess. Several abnormalities observed in this study were also observed in metabolomics profiling of airway epithelial cells from people with cystic fibrosis[80]. The predictive value and specificity of these compounds is deserving of prospective quantitative analysis in dogs predisposed to mucocele formation as compared to age and sex-matched control groups of dogs having non-mucocele-related causes of hepatobiliary disease.

### Insights into future medical strategies to ameliorate mucocele formation

Currently there are no evidence-based strategies for medical management of gallbladder mucocele formation. In this study, a number of compounds were identified as having potential therapeutic value in either restoring function to a vital metabolic pathway or in promoting normal secretory function of the gallbladder epithelium. Whether there is a clinically significant deficiency in any of these compounds will require individual quantitative analysis. If deficiency is documented, prospective studies to examine their ability to ameliorate gallbladder mucocele formation in dogs would be an exciting new direction for medical management.

### Study limitations

Significant limitations of this study include lack of inclusion of a group of dogs with other causes of gallbladder disease (such as cholecystitis or cholelithiasis) and a potential confounding influence of concurrent drug administration on results obtained from these clinical patients. Accordingly, some of our findings may also be observed in dogs with other causes of gallbladder disease. To minimize any impact of secondary cholestasis on our findings, only non-icteric dogs were included in the study among which only 3 dogs had mild increases in serum total bilirubin. Nonetheless, in order to validate these findings, a prospective study utilizing targeted and quantitative analysis of potential biomarker and therapeutic compounds will need to be conducted on normal dogs, dogs with suspected mucocele formation, and dogs with other causes of gallbladder or gastrointestinal disease. Concurrent drug administration in dogs with mucocele formation was unavoidable because hepatic duct bile samples could only be obtained from these patients while under general anesthesia for cholecystectomy. It was likewise impractical to recapitulate these circumstances in control dogs. Consequently, the presence of several compounds reflected their exogenous administration. This was noted for increases in pentobarbital, benzyl alcohol, and various drug preservatives in control dogs as they were sedated prior to serum collection and euthanized prior to collection of bile. Other limitations of the current study involve differences in the ages of dogs with mucocele formation compared to control dogs. It is possible that the Random Forest analysis identified some compounds simply on the basis of a difference in age or gender between the two groups of dogs. For example, increased erythritol and C-glycosyltryptophan in serum of dogs with mucocele formation likely reflects their higher chronological age compared to control dogs as has been recently demonstrated in aging humans[81]. Other confounding factors to consider are that dogs with mucocele formation had received a variety of commercial and home-cooked diets while the diet of control dogs was restricted to a dry research chow. Additionally, the duration of fasting prior to serum sample collection from dogs with mucocele formation was unknown but occurred consistently after fasting in control dogs. As with any global non-targeted metabolomics study, it is frequently not possible to determine the organ, cell type, or intracellular compartment from which compounds have originated. Moreover, the impact of renal excretion versus reabsorption and intestinal microbial metabolism on the types and quantity of compounds demonstrated in serum and bile remains unknown. Metabolomics investigation of both urine and intestinal content of dogs with mucocele formation compared to control dogs would provide considerable additional insight into the metabolic abnormalities identified in this study.

## Supporting information

S1 Methods (DOCX)

S1 Table Drugs to which dogs with mucocele formation were exposed at the time of collection of serum and/or hepatic duct bile. (DOCX)

S2 Table Compounds that were identified in either the serum or hepatic duct bile as significantly different (P<0.10) between control dogs and dogs with gallbladder mucocele formation. Bolded entries represent compounds and their origin (serum on left of table; hepatic duct bile on right of table) that were identified as significantly different (P<0.05) between control dogs and dogs with gallbladder mucocele formation. Superscript numbers, when present, indicate rank of the compound as identified by Random Forest analysis as able to distinguish between control dogs and dogs with gallbladder mucocele formation. % filled values represents the percentage of control and gallbladder mucocele dogs in which the compound was identified. Compounds with no numeric entries were not identified in the respective sample type.
(DOCX)
