# Supplementary material for: Qualitative metabolomics profiling of serum and bile from dogs with gallbladder mucocele formation
Source: PLoS One. 2018 Jan 11;13(1):e0191076. doi: 10.1371/journal.pone.0191076 (PMC5764353; doi:10.1371/journal.pone.0191076)
Supplement: S1 Methods — (DOCX) [file pone.0191076.s001.docx]

**S1 Methods:**

**Sample Accessioning:** Each sample received was accessioned into the Metabolon LIMS system and was assigned by the LIMS a unique identifier, which was associated with the original source identifier only. This identifier was used to track all sample handling, tasks, results *etc.* The samples (and all derived aliquots) were bar-coded and tracked by the LIMS system. All portions of any sample were automatically assigned their own unique identifiers by the LIMS when a new task was created; the relationship of these samples was also tracked. All samples were maintained at -80ºC until processed.

**Sample Preparation:** The sample preparation process was carried out using the automated MicroLab STAR® system from Hamilton Company. Recovery standards were added prior to the first step in the extraction process for QC purposes. Sample preparation was conducted using a proprietary series of organic and aqueous extractions to remove the protein fraction while allowing maximum recovery of small molecules. The resulting extract was divided into two fractions; one for analysis by UPLC-MS/MS and one for analysis by GC/MS. Samples were placed briefly on a TurboVap® (Zymark) to remove the organic solvent. Each sample was then frozen and dried under vacuum. Samples were then prepared for the appropriate instrument, either LC/MS or GC/MS.

**QA/QC:** For QA/QC purposes, a number of additional samples were included in each day’s analysis. Furthermore, a selection of QC compounds was added to every sample, including those under test. These compounds are carefully chosen so as not to interfere with the measurement of the endogenous compounds. Tables 1 and 2 describe the QC samples and compounds. These QC samples are primarily used to evaluate the process control for each study as well as aiding in the data curation.

**Table 1:** Description of Metabolon QC Samples

| **Type** | **Description** | **Purpose** |
| --- | --- | --- |
| MTRX | Large pool of human plasma maintained by Metabolon that has been characterized extensively. | Assure that all aspects of Metabolon process are operating within specifications. |
| CMTRX | Pool created by taking a small aliquot from every customer sample. | Assess the effect of a non-plasma matrix on the Metabolon process and distinguish biological variability from process variability. |
| PRCS | Aliquot of ultra-pure water | Process Blank used to assess the contribution to compound signals from the process. |
| SOLV | Aliquot of solvents used in extraction. | Solvent blank used to segregate contamination sources in the extraction. |

**Table 2:** Metabolon QC Standards

| **Type** | **Description** | **Purpose** |
| --- | --- | --- |
| DS | Derivatization Standard | Assess variability of derivatization for GC/MS samples. |
| IS | Internal Standard | Assess variability and performance of instrument. |
| RS | Recovery Standard | Assess variability and verify performance of extraction and instrumentation. |

**Liquid chromatography/Mass Spectrometry (LC/MS, LC/MS^2^**):  The LC/MS portion of the platform was based on a Waters ACQUITY UPLC and a Thermo-Finnigan LTQ mass spectrometer, which consisted of an electrospray ionization (ESI) source and linear ion-trap (LIT) mass analyzer.  The sample extract was split into two aliquots, dried, then reconstituted in acidic or basic LC-compatible solvents, each of which contained 11 or more injection standards at fixed concentrations. One aliquot was analyzed using acidic positive ion optimized conditions and the other using basic negative ion optimized conditions in two independent injections using separate dedicated columns. Extracts reconstituted in acidic conditions were gradient eluted using water and methanol both containing 0.1% formic acid, while the basic extracts, which also used water/methanol, contained 6.5mM Ammonium Bicarbonate. The MS analysis alternated between MS and data-dependent MS^2^ scans using dynamic exclusion, and the scan range was from 80-1000 *m/z*. Raw data files were archived and extracted as described below.

**Gas chromatography/Mass Spectrometry (GC/MS**):  The samples destined for GC/MS analysis were re-dried under vacuum desiccation for a minimum of 24 hours prior to being derivatized under dried nitrogen using bistrimethyl-silyl-triflouroacetamide (BSTFA).  The GC column was 5% phenyl and the temperature ramp is from 40° to 300° C in a 16 minute period.  Samples were analyzed on a Thermo-Finnigan Trace DSQ fast-scanning single-quadrupole mass spectrometer using electron impact ionization.  The instrument was tuned and calibrated for mass resolution and mass accuracy on a daily basis.  The information output from the raw data files was automatically extracted as discussed below.

**Data Extraction and Compound Identification:** Compounds were identified by comparison to library entries of purified standards or recurrent unknown entities. Metabolon maintains a library based on authenticated standards that contains the retention time/index (RI), mass to charge ratio (*m/z)*, and chromatographic data (including MS/MS spectral data) on all molecules present in the library. Furthermore, biochemical identifications are based on three criteria: retention index within a narrow RI window of the proposed identification, nominal mass match to the library +/- 0.4 amu, and the MS/MS forward and reverse scores between the experimental data and authentic standards. The MS/MS scores are based on a comparison of the ions present in the experimental spectrum to the ions present in the library spectrum. While there may be similarities between these molecules based on one of these factors, the use of all three data points can be utilized to distinguish and differentiate biochemicals. More than 3500 commercially available purified standard compounds have been acquired and registered into LIMS for distribution to both the LC-MS and GC-MS platforms for determination of their analytical characteristics.

**Curation:** A variety of curation procedures were carried out to ensure that a high quality data set was made available for statistical analysis and data interpretation. The QC and curation processes were designed to ensure accurate and consistent identification of true chemical entities, and to remove those representing system artifacts, mis-assignments, and background noise. Data analysts use proprietary visualization and interpretation software to confirm the consistency of peak identification among the various samples. Library matches for each compound were checked for each sample and corrected if necessary.
