# Supplementary material for: Qualitative metabolomics profiling of serum and bile from dogs with gallbladder mucocele formation
Source: PLoS One. 2018 Jan 11;13(1):e0191076. doi: 10.1371/journal.pone.0191076 (PMC5764353; doi:10.1371/journal.pone.0191076)
Supplement: S1 Table — (DOCX) [file pone.0191076.s002.docx]

| **Category and identity of drugs** | **Number (%) of dogs** |
| --- | --- |
| **Any anesthetic and preanesthetic** | **10 (100%)** |
| Isofluorane | 10 (100%) |
| Propofol | 9 (90%) |
| Midazolam | 4 (40%) |
| Etomidate | 1 (10%) |
| **Any opioid analgesic** | **10 (100%)** |
| Fentanyl | 8 (80%) |
| Hydromorphone | 6 (60%) |
| Buprenorphine | 3 (30%) |
| Butorphanol | 2 (20%) |
| Morphine | 2 (20%) |
| Tramadol | 1 (10%) |
| **Any antibiotic** | **10 (100%)** |
| Cefazolin | 7 (70%) |
| Ampicillin-sulbactam | 3 (30%) |
| Amoxicillin-clavulonate | 2 (20%) |
| Amoxicillin, enrofloxacin, or metronidazole | 1 (10%) |
| **Any adrenergic or cholinergic** | **7 (70%)** |
| Glycopyrrolate | 5 (50%) |
| Dopamine | 3 (30%) |
| Acepromazine, or atenolol | 1 (10%) |
| **Any flea, tick, and heartworm preventative** | **7 (70%)** |
| Fipronil | 6 (60%) |
| Milbemycin | 5 (50%) |
| Ivermectin | 2 (20%) |
| Methylsulfonomethane | 2 (20%) |
| Dinotefuran, imidacloprid, methoprene, nitenpyram,  permethrin, pyrantel, or pyriproxyfen | 1 (10%) |
| **Any vitamin or joint supplement** | **6 (60%)** |
| Vitamin K | 5 (50%) |
| Glucosamine | 2 (20%) |
| Chondroitin sulfate, multivitamin, or vitamin B12 | 1 (10%) |
| **Any colloid** | **4 (40%)** |
| Hetastarch | 2 (20%) |
| Voluven | 2 (20%) |
| **Any antiemetic or gastroprotective** | **3 (30%)** |
| Maropitant | 2 (20%) |
| Famotidine, omeprazole, ondansetron, pantoprazole, or  sucralfate | 1 (10%) |
| **Any hepatic support** | **3 (30%)** |
| Ursodeoxycholic acid | 2 (20%) |
| S-adenosyl methionine, silibin, colchicine, or lactulose | 1 (10%) |
| **Any endocrine** | **3 (30%)** |
| Dexamethasone, prednisone, thyroxine, or trilostane | 1 (10%) |
| **Any cardiac** | **2 (20%)** |
| Lidocaine | 2 (20%) |
| Bupivocaine, enalapril, furosemide, or pimobendan | 1 (10%) |

**S1 Table.** Drugs to which dogs with mucocele formation were exposed at the time of collection of serum and/or hepatic duct bile.
