# Supplementary material for: Qualitative metabolomics profiling of serum and bile from dogs with gallbladder mucocele formation
Source: PLoS One. 2018 Jan 11;13(1):e0191076. doi: 10.1371/journal.pone.0191076 (PMC5764353; doi:10.1371/journal.pone.0191076)
Supplement: S2 Table — Bolded entries represent compounds and their origin (serum on left of table; hepatic duct bile on right of table) that were identified as significantly different (P<0.05) between control dogs and dogs with gallbladder mucocele formation. Superscript numbers, when present, indicate rank of the compound as identified by Random Forest analysis as able to distinguish between control dogs and dogs with gallbladder mucocele formation. % filled values represents the percentage of control and gallbladder mucocele dogs in which the compound was identified. Compounds with no numeric entries were not identified in the respective sample type. (DOCX) [file pone.0191076.s003.docx]

**S2 Table.** Compounds that were identified in either the serum or hepatic duct bile as significantly different (P<0.10) between control dogs and dogs with gallbladder mucocele formation. Bolded entries represent compounds and their origin (serum on left of table; hepatic duct bile on right of table) that were identified as significantly different (P<0.05) between control dogs and dogs with gallbladder mucocele formation. Superscript numbers, when present, indicate rank of the compound as identified by Random Forest analysis as able to distinguish between control dogs and dogs with gallbladder mucocele formation. % filled values represents the percentage of control and gallbladder mucocele dogs in which the compound was identified. Compounds with no numeric entries were not identified in the respective sample type.

| **Amino Acids** | **Biochemical Name** | **Fold Change in Serum** | **Welch's Two-Sample *t*-Test** | **Mean Values** | | **% Filled Values** | | **Biochemical Name** | **Fold Change in Bile** | **Welch's Two-Sample *t*-Test** | **Mean Values** | | **% Filled Values** | |
| --- | --- | --- | --- | --- | --- | --- | --- | --- | --- | --- | --- | --- | --- | --- |
|  |  |  |  |  |  |  |  |  |  |  |  |  |  |  |
|  |  |  | **p-value** | **No GBM** | **GBM** | **No GBM** | **GBM** |  |  | **p-value** | **No GBM** | **GBM** | **No GBM** | **GBM** |
| Aliphatic and Branch Chained Amino Acids | **gamma-glutamylvaline^1^** | **2.25** | **6.34E-06** | 0.6813 | 1.5317 | 100 | 100 | gamma-glutamylvaline |  |  |  |  |  |  |
|  | **gamma-glutamylisoleucine^4^** | **1.88** | **2.17E-05** | 0.7155 | 1.3436 | 100 | 100 | gamma-glutamylisoleucine |  |  |  |  |  |  |
|  | **gamma-glutamylleucine** | **1.63** | **0.0019** | 0.8709 | 1.4217 | 100 | 100 | gamma-glutamylleucine |  |  |  |  |  |  |
|  | **N-acetylalanine** | **1.45** | **0.0095** | 0.8418 | 1.2244 | 90 | 90 | N-acetylalanine | -1.02 | 0.2734 | 1.1314 | 1.111 | 100 | 90 |
|  | **dimethylglycine** | **1.48** | **0.0192** | 0.8716 | 1.287 | 90 | 100 | dimethylglycine | 1.21 | 0.2441 | 0.9648 | 1.1716 | 90 | 100 |
|  | **N-acetylglycine** | **1.53** | **0.0281** | 0.8651 | 1.3228 | 100 | 100 | N-acetylglycine |  |  |  |  |  |  |
|  | **allo-isoleucine** | **1.46** | **0.0418** | 0.8708 | 1.2703 | 100 | 100 | allo-isoleucine |  |  |  |  |  |  |
|  | N-acetylleucine | 1.94 | 0.0756 | 0.7618 | 1.4799 | 100 | 100 | **N-acetylleucine^6^** | **-3.03** | **0.0002** | 1.5544 | 0.5119 | 100 | 100 |
|  | N-acetylisoleucine | 1.86 | 0.0725 | 0.8033 | 1.492 | 100 | 100 | **N-acetylisoleucine** | **-2.38** | **0.0004** | 1.5142 | 0.6354 | 100 | 100 |
|  | N-acetylvaline |  |  |  |  |  |  | **N-acetylvaline** | **-2.13** | **0.0004** | 1.2706 | 0.5971 | 100 | 50 |
|  | 3-methyl-2-oxobutyrate | 1.32 | 0.1757 | 1.0339 | 1.3633 | 100 | 100 | **3-methyl-2-oxobutyrate** | **4.21** | **0.0075** | 0.249 | 1.0483 | 20 | 70 |
|  | isovalerylcarnitine | 1.76 | 0.0682 | 0.7604 | 1.3387 | 100 | 100 | **isovalerylcarnitine** | **2.52** | **0.0136** | 0.765 | 1.9264 | 60 | 90 |
|  | isoleucine | 1.14 | 0.198 | 0.9524 | 1.0888 | 100 | 100 | **isoleucine** | **6.06** | **0.0473** | 1.1935 | 7.229 | 90 | 100 |
|  | leucine | 1.16 | 0.1631 | 0.9557 | 1.1129 | 100 | 100 | leucine | 8.84 | 0.0504 | 1.2334 | 10.9059 | 90 | 100 |
|  | valine | 1.15 | 0.1806 | 0.9227 | 1.0651 | 100 | 100 | valine | 4.86 | 0.0518 | 1.1693 | 5.6836 | 90 | 100 |
|  | 2-methylbutyrylcarnitine (C5) | 1.40 | 0.2687 | 0.8621 | 1.2067 | 100 | 100 | 2-methylbutyrylcarnitine (C5) | 1.56 | 0.0831 | 0.8557 | 1.3362 | 40 | 90 |
|  | 3-methylglutarylcarnitine (C6) | 3.22 | 0.053 | 0.6517 | 2.097 | 60 | 80 | 3-methylglutarylcarnitine (C6) | -1.25 | 0.5484 | 1.2818 | 1.0286 | 100 | 80 |
| Hydroxyl Amino Acids | **N-acetylthreonine** | **1.92** | **0.0029** | 0.6204 | 1.1901 | 50 | 90 | **N-acetylthreonine** | **-1.96** | **0.0389** | 1.8009 | 0.9253 | 90 | 90 |
|  | **threonine** | **-1.69** | **0.023** | 1.0482 | 0.6208 | 90 | 70 | threonine | 12.37 | 0.1349 | 0.9347 | 11.5601 | 90 | 100 |
|  | N-acetylserine | 1.75 | 0.0615 | 0.8429 | 1.4769 | 100 | 100 | N-acetylserine |  |  |  |  |  |  |
|  | serine | -1.23 | 0.1161 | 1.0964 | 0.8928 | 100 | 100 | serine | 27.98 | 0.0815 | 1.3215 | 36.9778 | 90 | 100 |
|  | beta-hydroxypyruvate | 1.08 | 0.5609 | 0.9593 | 1.0355 | 100 | 100 | **beta-hydroxypyruvate** | **-2.00** | **0.0336** | 1.0435 | 0.5261 | 80 | 50 |
| Acidic Amino Acids | **glutamate^3^** | **3.51** | **0.0005** | 0.7384 | 2.5887 | 100 | 100 | glutamate | 1.70 | 0.9572 | 1.1905 | 2.0296 | 100 | 100 |
|  | **N-acetylglutamine** | **1.35** | **0.0331** | 0.8932 | 1.21 | 100 | 100 | **N-acetylglutamine** | **-2.56** | **0.0007** | 1.3025 | 0.5081 | 100 | 100 |
|  | glutamine | -1.19 | 0.0513 | 1.0993 | 0.9247 | 100 | 100 | glutamine | 2.35 | 0.0604 | 0.8421 | 1.9792 | 90 | 100 |
|  | pyroglutamine | 1.20 | 0.5704 | 1.1102 | 1.3363 | 100 | 100 | **pyroglutamine** | **-2.44** | **0.0163** | 2.1093 | 0.8628 | 100 | 100 |
|  | asparagine | 1.16 | 0.6592 | 1.0149 | 1.1795 | 100 | 100 | **asparagine** | **30.83** | **0.029** | 0.274 | 8.4485 | 0 | 80 |
|  | aspartate |  |  |  |  |  |  | **aspartate** | **59.51** | **0.0636** | 0.9935 | 59.1238 | 90 | 100 |

| Basic Amino Acids | **lysine** | **1.46** | **0.0045** | 0.8758 | 1.2813 | 100 | 100 | **lysine** | **9.79** | **0.0312** | 0.7636 | 7.4769 | 60 | 100 |
| --- | --- | --- | --- | --- | --- | --- | --- | --- | --- | --- | --- | --- | --- | --- |
|  | **N-acetyl-1-methylhistidine^27^** | **11.72** | **0.013** | 0.6903 | 8.09 | 80 | 90 | N-acetyl-1-methylhistidine | 2.44 | 0.1229 | 0.827 | 2.0211 | 100 | 100 |
|  | **N-acetyl-3-methylhistidine** | **6.34** | **0.0137** | 0.6541 | 4.1462 | 60 | 90 | N-acetyl-3-methylhistidine |  |  |  |  |  |  |
|  | **N-acetylhistidine** | **1.80** | **0.0183** | 0.7988 | 1.4389 | 60 | 80 | **N-acetylhistidine** | **-2.94** | **0.0015** | 1.4256 | 0.4796 | 100 | 90 |
|  | **3-methylhistidine** | **3.18** | **0.0404** | 0.932 | 2.9601 | 100 | 100 | 3-methylhistidine |  |  |  |  |  |  |
|  | imidazole lactate | 2.24 | 0.084 | 0.5555 | 1.2418 | 50 | 60 | imidazole lactate |  |  |  |  |  |  |
|  | arginine | -1.22 | 0.0992 | 1.0534 | 0.8688 | 100 | 100 | arginine | 2.61 | 0.3684 | 1.2869 | 3.3538 | 100 | 100 |
|  | histidine | 1.02 | 0.9823 | 0.9542 | 0.9738 | 100 | 100 | **histidine** | **5.92** | **0.0141** | 0.3517 | 2.0831 | 30 | 90 |
|  | carnosine | 1.31 | 0.1366 | 0.8849 | 1.1623 | 100 | 100 | **carnosine** | **-1.89** | **0.0241** | 1.2359 | 0.6494 | 100 | 100 |
|  | anserine | 1.09 | 0.655 | 0.9807 | 1.0666 | 100 | 100 | **anserine** | **-2.08** | **0.0025** | 1.2872 | 0.6227 | 100 | 90 |
|  | homocarnosine |  |  |  |  |  |  | **homocarnosine** | **-2.63** | **0.0145** | 1.8396 | 0.6972 | 100 | 90 |
|  | trans-urocanate | 1.45 | 0.2608 | 0.9198 | 1.3317 | 100 | 100 | **trans-urocanate** | **-2.94** | **0.0372** | 3.0151 | 1.0306 | 100 | 90 |
|  | N2-acetyllysine |  |  |  |  |  |  | **N2-acetyllysine** | **-3.13** | **0.0061** | 1.5789 | 0.5094 | 90 | 70 |
|  | imidazole propionate | 2.75 | 0.9615 | 0.9781 | 2.6934 | 100 | 100 | **imidazole propionate** | **-3.33** | **0.032** | 2.9565 | 0.8951 | 100 | 100 |
|  | 5-aminovalerate |  |  |  |  |  |  | **5-aminovalerate** | **-3.33** | **0.0356** | 2.3363 | 0.6962 | 90 | 90 |
| Aromatic Amino Acids | **gamma-glutamylphenylalanine^26^** | **1.63** | **0.0041** | 0.8223 | 1.3376 | 100 | 100 | gamma-glutamylphenylalanine |  |  |  |  |  |  |
|  | **N-acetyltryptophan** | **2.97** | **0.0054** | 0.9173 | 2.7249 | 100 | 100 | **N-acetyltryptophan^1^** | **-5.56** | **1.01E-05** | 3.4352 | 0.6065 | 100 | 90 |
|  | **phenyllactate (PLA)^28^** | **1.93** | **0.0071** | 0.7573 | 1.4584 | 60 | 90 | phenyllactate (PLA) |  |  |  |  |  |  |
|  | **C-glycosyltryptophan^9^** | **2.38** | **0.0084** | 0.8058 | 1.9207 | 100 | 100 | **C-glycosyltryptophan** | **-2.17** | **0.018** | 1.249 | 0.5723 | 80 | 70 |
|  | **quinaldic acid** | **-6.67** | **0.0131** | 4.3186 | 0.6685 | 100 | 40 | **quinaldic acid** | **-4.35** | **0.0005** | 2.5626 | 0.5817 | 100 | 90 |
|  | **indolelactate** | **5.33** | **0.0149** | 0.8595 | 4.5777 | 100 | 100 | **indolelactate** | **-1.75** | **0.031** | 1.4711 | 0.8314 | 100 | 90 |
|  | **gamma-glutamyltyrosine** | **1.24** | **0.0345** | 0.8753 | 1.082 | 100 | 100 | gamma-glutamyltyrosine |  |  |  |  |  |  |
|  | indolepyruvate | 1.93 | 0.056 | 0.4409 | 0.8514 | 0 | 50 | indolepyruvate |  |  |  |  |  |  |
|  | phenylalanine | 1.22 | 0.0712 | 1.0002 | 1.2186 | 100 | 100 | **phenylalanine** | **7.98** | **0.0402** | 1.1365 | 9.0673 | 100 | 100 |
|  | serotonin (5HT) | -1.61 | 0.0942 | 1.2426 | 0.7761 | 100 | 100 | serotonin (5HT) |  |  |  |  |  |  |
|  | 3-(4-hydroxyphenyl)lactate | 1.88 | 0.1002 | 0.9498 | 1.7861 | 100 | 100 | **3-(4-hydroxyphenyl)lactate** | **-2.00** | **0.0124** | 1.7764 | 0.8911 | 100 | 100 |
|  | phenylacetylglutamine | 1.98 | 0.1653 | 0.9483 | 1.8733 | 70 | 90 | **phenylacetylglutamine** | **-2.00** | **0.0122** | 2.2723 | 1.1341 | 100 | 100 |
|  | 4-hydroxyphenylacetyl glycine | 1.53 | 0.4487 | 0.9352 | 1.431 | 60 | 70 | **4-hydroxyphenylacetyl glycine** | **1.22** | **0.03** | 2.2404 | 2.7266 | 100 | 100 |
|  | kynurenate | -1.08 | 0.4511 | 0.9622 | 0.8921 | 100 | 100 | **kynurenate^16^** | **-4.17** | **0.003** | 1.5312 | 0.3649 | 100 | 100 |
|  | tryptophan | 1.15 | 0.476 | 1.0547 | 1.2151 | 100 | 100 | **tryptophan** | **3.69** | **0.0323** | 1.0937 | 4.038 | 70 | 100 |
|  | tyrosine | -1.03 | 0.6461 | 1.0245 | 0.9907 | 100 | 100 | tyrosine | 11.75 | 0.0578 | 0.8971 | 10.5369 | 80 | 100 |
|  | phenylacetylglycine | 1.44 | 0.7302 | 0.8839 | 1.2744 | 90 | 100 | **phenylacetylglycine** | **-1.79** | **0.0208** | 1.9054 | 1.06 | 100 | 100 |
|  | N-acetyltyrosine |  |  |  |  |  |  | **N-acetyltyrosine^19^** | **-3.70** | **0.0073** | 2.0396 | 0.5448 | 100 | 30 |
|  | 3-[3-(sulfooxy)phenyl]propanoic acid |  |  |  |  |  |  | **3-[3-(sulfooxy)phenyl]propanoic acid^29^** | **-20.00** | **0.0012** | 2.6203 | 0.1276 | 90 | 0 |
|  | indoleacetylglutamine |  |  |  |  |  |  | **indoleacetylglutamine** | **-2.44** | **0.0142** | 1.6653 | 0.6805 | 100 | 90 |
| Cyclic Amino Acid | proline | 1.03 | 0.778 | 1.0269 | 1.0563 | 100 | 100 | proline | 6.94 | 0.0585 | 0.7275 | 5.0511 | 100 | 100 |
|  | trans-4-hydroxyproline | -1.37 | 0.273 | 1.6646 | 1.2206 | 100 | 100 | trans-4-hydroxyproline | 1.77 | 0.0844 | 0.7178 | 1.2724 | 40 | 90 |
| Creatine Metabolism | creatine | 1.32 | 0.4252 | 0.8868 | 1.1699 | 100 | 100 | **creatine** | **-2.00** | **0.0371** | 1.738 | 0.8715 | 100 | 100 |
|  | creatinine | 1.23 | 0.5191 | 1.0033 | 1.2384 | 100 | 100 | **creatinine^13^** | **-7.69** | **0.0002** | 3.7128 | 0.4871 | 100 | 100 |
| Urea cycle | **homocitrulline** | **3.98** | **0.0031** | 0.8233 | 3.2745 | 100 | 100 | homocitrulline | 1.06 | 0.6549 | 2.1276 | 2.2506 | 80 | 90 |
|  | **ornithine** | **1.77** | **0.0311** | 0.8907 | 1.5783 | 100 | 100 | ornithine | 2.21 | 0.479 | 0.8903 | 1.9681 | 90 | 100 |
| Polyamine Metabolism | putresine |  |  |  |  |  |  | **putrescine^25^** | **-8.33** | **0.0009** | 3.364 | 0.387 | 90 | 70 |
| Felinine Metabolism | N-acetylfelinine | 1.21 | 0.8617 | 1.2489 | 1.5108 | 100 | 90 | **N-acetylfelinine** | **-9.09** | **0.0018** | 3.5924 | 0.3937 | 90 | 70 |
| Sulfur Amino Acids | **N-formylmethionine** | **1.53** | **0.0197** | 0.8816 | 1.345 | 100 | 80 | N-formylmethionine | -1.14 | 0.3009 | 0.913 | 0.8018 | 100 | 60 |
|  | **N-acetylmethionine** | **1.43** | **0.0283** | 0.8646 | 1.2352 | 100 | 90 | N-acetylmethionine | 1.00 | 0.1449 | 1.1981 | 1.1977 | 100 | 100 |
|  | **gamma-glutamylmethionine** | **-1.47** | **0.0366** | 1.2311 | 0.8423 | 100 | 100 | gamma-glutamylmethionine |  |  |  |  |  |  |
|  | S-methylcysteine | 1.36 | 0.0678 | 0.8595 | 1.1661 | 100 | 100 | S-methylcysteine | 1.15 | 0.5512 | 0.9421 | 1.0856 | 50 | 60 |
|  | methionine | -1.11 | 0.244 | 1.0231 | 0.9235 | 100 | 100 | methionine | 5.57 | 0.056 | 0.9156 | 5.0966 | 100 | 100 |
|  | cysteine s-sulfate |  |  |  |  |  |  | cysteine s-sulfate | 25.69 | 0.108 | 0.5151 | 13.2344 | 40 | 50 |
|  | taurine |  |  |  |  |  |  | **taurine^9^** | **-20.00** | **7.70E-05** | 2.9211 | 0.1495 | 90 | 90 |
| Dipeptide | **glycylglycine** | **2.33** | **0.0013** | 0.7024 | 1.6339 | 100 | 100 | glycylglycine |  |  |  |  |  |  |
|  | isoleucylglutamine |  |  |  |  |  |  | **isoleucylglutamine** | **8.10** | **0.0491** | 0.47 | 3.8084 | 30 | 60 |
|  | aspartylleucine |  |  |  |  |  |  | aspartylleucine | 4.39 | 0.0601 | 0.1759 | 0.7723 | 0 | 50 |
|  | phenylalanylaspartate |  |  |  |  |  |  | phenylalanylaspartate | 5.10 | 0.0637 | 0.13 | 0.6626 | 0 | 50 |
|  | threonylalanine |  |  |  |  |  |  | threonylalanine | 3.71 | 0.0769 | 0.336 | 1.2462 | 10 | 40 |
|  | glycylproline |  |  |  |  |  |  | glycylproline | 3.17 | 0.0813 | 0.2423 | 0.7686 | 10 | 40 |
|  | alanylalanine |  |  |  |  |  |  | alanylalanine | 7.84 | 0.0845 | 0.1253 | 0.9829 | 10 | 40 |
|  | tyrosylglutamate |  |  |  |  |  |  | tyrosylglutamate | 5.09 | 0.0856 | 0.1843 | 0.938 | 20 | 40 |
|  | valylglycine |  |  |  |  |  |  | valylglycine | 11.76 | 0.086 | 0.0477 | 0.561 | 0 | 40 |
|  | prolylglutamate |  |  |  |  |  |  | prolylglutamate | 2.68 | 0.0969 | 0.2261 | 0.6049 | 10 | 30 |
|  | tyrosylglutamine |  |  |  |  |  |  | tyrosylglutamine | 4.24 | 0.0986 | 0.1745 | 0.74 | 10 | 30 |
|  | valylglutamate |  |  |  |  |  |  | valylglutamate | 4.86 | 0.0992 | 0.9877 | 4.8049 | 40 | 70 |

| **Redox** | **Biochemical Name** | **Fold Change in Serum** | **Welch's Two-Sample *t*-Test** | **Mean Values** | | **% Filled Values** | | **Biochemical Name** | **Fold Change in Bile** | **Welch's Two-Sample *t*-Test** | **Mean Values** | | **% Filled Values** | |
| --- | --- | --- | --- | --- | --- | --- | --- | --- | --- | --- | --- | --- | --- | --- |
|  |  |  | **p-value** | **No GBM** | **GBM** | **No GBM** | **GBM** |  |  | **p-value** | **No GBM** | **GBM** | **No GBM** | **GBM** |
|  | cys-gly, oxidized |  |  |  |  |  |  | **cys-gly, oxidized^10^** | **-50.0** | **2.49E-05** | 2.2858 | 0.0507 | 90 | 50 |
|  | cysteinylglycine |  |  |  |  |  |  | **cysteinylglycine** | **-5.3** | **0.017** | 3.5451 | 0.6748 | 90 | 90 |
|  | glutathione, oxidized (GSSG) | -1.69 | 0.088 | 1.855 | 1.0973 | 100 | 50 | glutathione, oxidized (GSSG) | 2.2 | 0.810 | 1.8767 | 4.126 | 50 | 60 |
|  | **cysteine-glutathione disulfide^18^** | **-3.70** | **3.00E-04** | 1.3045 | 0.3477 | 100 | 70 | cysteine-glutathione disulfide | -1.6 | 0.459 | 1.9753 | 1.2283 | 100 | 80 |
|  | ascorbate (Vitamin C) | -2.94 | 0.200 | 2.3015 | 0.7778 | 100 | 90 | **ascorbate (Vitamin C)^20^** | **-16.7** | **0.002** | 7.4042 | 0.4349 | 100 | 70 |
|  | 2-ketogulonate |  |  |  |  |  |  | **2-ketogulonate^4^** | **-4.8** | **0.0001** | 2.5184 | 0.5213 | 100 | 100 |
|  | gulono-1,4-lactone | 1.6 | 0.322 | 1.0294 | 1.6457 | 100 | 100 | **gulono-1,4-lactone** | **-2.2** | **0.018** | 1.8032 | 0.8048 | 100 | 100 |
|  | **alpha-tocopherol^16^** | **2.4** | **4.13E-05** | 0.6998 | 1.6774 | 100 | 100 | alpha-tocopherol | -1.3 | 0.162 | 1.0885 | 0.8341 | 100 | 100 |
|  | dihydrobiopterin |  |  |  |  |  |  | **dihydrobiopterin** | **-5.0** | **0.001** | 1.8249 | 0.3709 | 100 | 30 |
|  | isoxanthopterin |  |  |  |  |  |  | **isoxanthopterin** | **-4.0** | **0.027** | 1.1423 | 0.2861 | 80 | 60 |

| **Carbohydrates** | **Biochemical Name** | **Fold Change in Serum** | **Welch's Two-Sample *t*-Test** | **Mean Values** | | **% Filled Values** | | **Biochemical Name** | **Fold Change in Bile** | **Welch's Two-Sample *t*-Test** | **Mean Values** | | **% Filled Values** | |
| --- | --- | --- | --- | --- | --- | --- | --- | --- | --- | --- | --- | --- | --- | --- |
|  |  |  | **p-value** | **No GBM** | **GBM** | **No GBM** | **GBM** |  |  | **p-value** | **No GBM** | **GBM** | **No GBM** | **GBM** |
| Glycolysis, Gluconeogenesis, and Pyruvate Metabolism | pyruvate | 0.99 | 0.950 | 1.4827 | 1.4666 | 100 | 100 | **pyruvate** | **5.79** | **0.031** | 1.0022 | 5.7997 | 100 | 100 |
|  | **glycerate^8^** | **2.92** | **2.00E-04** | 0.6771 | 1.9771 | 100 | 100 | **glycerate** | **2.22** | **0.015** | 0.8975 | 1.994 | 100 | 100 |
|  | 1,5-anhydroglucitol (1,5-AG) | 1.16 | 0.259 | 0.9454 | 1.0925 | 100 | 100 | 1,5-anhydroglucitol (1,5-AG) | 1.69 | 0.053 | 0.9025 | 1.5238 | 100 | 100 |
| Tricarboxylic Acid Cycle | **citrate** | **1.42** | **0.029** | 0.8826 | 1.2562 | 100 | 100 | **citrate** | **3.47** | **0.016** | 0.7971 | 2.7679 | 100 | 100 |
|  | **succinate** | **1.28** | **0.042** | 0.853 | 1.0923 | 100 | 100 | **succinate^26^** | **-33.3** | **0.010** | 30.9225 | 0.7973 | 100 | 100 |
|  | **fumarate** | **1.63** | **0.003** | 0.7933 | 1.2906 | 100 | 100 | **fumarate** | **-2.27** | **0.006** | 1.332 | 0.5843 | 100 | 100 |
| Oxidative Phosphorylation | acetylphosphate |  |  |  |  |  |  | **acetylphosphate** | **-2.63** | **0.021** | 2.1647 | 0.8235 | 100 | 100 |
|  | pyrophosphate (PPi) |  |  |  |  |  |  | **pyrophosphate (PPi)** | **-2.56** | **0.030** | 2.1243 | 0.8338 | 100 | 100 |
|  | **phosphate^25^** | **1.55** | **0.001** | 0.7977 | 1.2384 | 100 | 100 | phosphate | -1.49 | 0.069 | 1.2153 | 0.813 | 100 | 100 |
| Nicotinamide | nicotinamide riboside |  |  |  |  |  |  | **nicotinamide riboside** | **-10.0** | **0.011** | 6.0066 | 0.5989 | 90 | 70 |
|  | nicotinamide | 1.37 | 0.818 | 1.0561 | 1.4432 | 100 | 100 | nicotinamide | 1.9 | 0.053 | 0.5866 | 1.1399 | 60 | 80 |
| Vitamin B2 | riboflavin |  |  |  |  |  |  | **riboflavin^14^** | **-5.0** | **0.007** | 1.899 | 0.3809 | 100 | 90 |
| Pantothenate and Coenzyme A | pantothenate | 1.09 | 0.913 | 1.0457 | 1.1443 | 100 | 100 | **pantothenate^23^** | **-3.2** | **0.002** | 1.377 | 0.4322 | 100 | 100 |
| Sugar Alcohol Metabolism | **ribose^5^** | **5.95** | **1.23E-06** | 0.4246 | 2.5267 | 100 | 100 | ribose | 2.4 | 0.209 | 1.0249 | 2.4554 | 100 | 100 |
|  | **threitol** | **2.61** | **0.009** | 0.8135 | 2.1207 | 100 | 100 | **threitol** | **-2.38** | **0.006** | 1.9911 | 0.8303 | 100 | 100 |
|  | **erythritol^23^** | **2.44** | **0.0117** | 0.8781 | 2.1443 | 100 | 100 | **erythritol** | **-2.63** | **0.0483** | 2.2453 | 0.8505 | 100 | 100 |
|  | **xylonate** | **2.05** | **0.001** | 0.6897 | 1.413 | 100 | 100 | **xylonate** | **-2.08** | **0.041** | 1.8233 | 0.8799 | 100 | 100 |
|  | arabitol | 1.54 | 0.452 | 1.1308 | 1.7435 | 100 | 100 | **arabitol** | **-4.35** | **0.013** | 2.4726 | 0.5768 | 100 | 90 |
|  | xylitol | 1.22 | 0.333 | 0.945 | 1.1499 | 100 | 100 | **xylitol** | **-2.44** | **0.005** | 1.8239 | 0.7448 | 100 | 100 |
|  | arabinose | -2.0 | 0.082 | 0.8196 | 0.4126 | 70 | 20 | arabinose |  |  |  |  |  |  |
|  | mannitol | 1.79 | 0.175 | 1.3071 | 2.3393 | 100 | 100 | **mannitol^2^** | **-6.25** | **2.00E-04** | 2.607 | 0.428 | 100 | 80 |
|  | sorbitol | 0.82 | 0.113 | 1.2333 | 1.0104 | 100 | 100 | sorbitol | -1.85 | 0.060 | 2.05 | 1.1167 | 100 | 100 |
| Aminosugar Metabolism | **erythronate** | **2.57** | **0.015** | 0.8305 | 2.1353 | 100 | 100 | erythronate | 0.44 | 0.980 | 2.8413 | 1.237 | 100 | 100 |
|  | **glucuronate** | **2.02** | **0.007** | 0.7144 | 1.4435 | 100 | 100 | glucuronate | 0.84 | 0.141 | 1.2916 | 1.0851 | 100 | 100 |
|  | **N-acetylneuraminate** | **1.79** | **0.011** | 0.8878 | 1.5866 | 100 | 100 | N-acetylneuraminate |  |  |  |  |  |  |
| Advanced Glycation End-product | erythrulose |  |  |  |  |  |  | **erythrulose** | **-2.13** | **0.044** | 1.7252 | 0.808 | 100 | 90 |

| **Nucleotides** | **Biochemical Name** | **Fold Change in Serum** | **Welch's Two-Sample *t*-Test** | **Mean Values** | | **% Filled Values** | | **Biochemical Name** | **Fold Change in Bile** | **Welch's Two-Sample *t*-Test** | **Mean Values** | | **% Filled Values** | |
| --- | --- | --- | --- | --- | --- | --- | --- | --- | --- | --- | --- | --- | --- | --- |
|  |  |  | **p-value** | **No GBM** | **GBM** | **No GBM** | **GBM** |  |  | **p-value** | **No GBM** | **GBM** | **No GBM** | **GBM** |
| Purine Metabolism | inosine | -1.37 | 0.0965 | 1.0241 | 0.7514 | 100 | 80 | inosine | 1.34 | 0.5795 | 1.1622 | 1.557 | 100 | 100 |
|  | xanthosine |  |  |  |  |  |  | **xanthosine** | **-5.26** | **0.0169** | 1.8105 | 0.3518 | 70 | 10 |
|  | 1-methylurate |  |  |  |  |  |  | **1-methylurate** | **-2.04** | **0.0042** | 1.2222 | 0.5968 | 90 | 60 |
|  | **adenosine 5'-monophosphate (AMP)^6^** | **-33.3** | **1.24E-05** | 1.2305 | 0.0343 | 100 | 20 | adenosine 5'-monophosphate (AMP) |  |  |  |  |  |  |
|  | **adenosine^10^** | **-16.7** | **0.0063** | 5.0533 | 0.3244 | 90 | 10 | **adenosine** | **-2.27** | **0.0216** | 1.441 | 0.6287 | 100 | 90 |
|  | adenosine 3',5'-cyclic monophosphate (cAMP) |  |  |  |  |  |  | **adenosine 3',5'-cyclic monophosphate (cAMP)^30^** | **-5.55** | **0.0003** | 1.2947 | 0.2269 | 90 | 70 |
|  | adenine |  |  |  |  |  |  | **adenine** | **-2.63** | **0.0041** | 1.5708 | 0.6038 | 100 | 100 |
|  | N6-carbamoylthreonyladenosine | 1.62 | 0.1919 | 0.9161 | 1.4831 | 100 | 100 | **N6-carbamoylthreonyladenosine** | **-3.03** | **0.0052** | 1.6692 | 0.5551 | 90 | 100 |
|  | **N2,N2-dimethylguanosine^13^** | **1.87** | **0.0057** | 0.8866 | 1.658 | 100 | 100 | **N2,N2-dimethylguanosine** | **-2.7** | **0.019** | 1.557 | 0.5795 | 100 | 100 |
|  | **guanosine^21^** | **-2.13** | **0.0002** | 1.2735 | 0.6024 | 100 | 50 | guanosine | 2.36 | 0.3718 | 0.8258 | 1.9525 | 50 | 80 |
|  | 7-methylguanosine |  |  |  |  |  |  | **7-methylguanosine** | **-6.25** | **0.0344** | 1.476 | 0.2347 | 60 | 40 |
|  | N1-methylguanosine |  |  |  |  |  |  | **N1-methylguanosine** | **-3.03** | **0.0075** | 1.6562 | 0.5465 | 90 | 90 |
|  | 7-methylguanine | 1.0 | 0.8366 | 1.0315 | 1.0365 | 100 | 100 | 7-methylguanine | -2.5 | 0.097 | 2.4384 | 0.9669 | 100 | 90 |
| Pyrimidine Metabolism | dihydroorotate |  |  |  |  |  |  | **dihydroorotate** | **-3.7** | **0.0206** | 1.586 | 0.4331 | 90 | 100 |
|  | orotate |  |  |  |  |  |  | **orotate** | **-3.12** | **0.0172** | 2.4771 | 0.8028 | 100 | 90 |
|  | **uridine** | **-2.38** | **0.0209** | 1.6777 | 0.6963 | 100 | 70 | **uridine** | **1.94** | **0.0095** | 1.0012 | 1.9446 | 100 | 100 |
|  | **uracil^2^** | **4.89** | **8.99E-07** | 0.5371 | 2.6288 | 100 | 100 | uracil | -1.12 | 0.2068 | 2.0596 | 1.839 | 90 | 100 |
|  | **3-ureidopropionate** | **2.4** | **0.0011** | 0.7697 | 1.8435 | 100 | 100 | 3-ureidopropionate | -2.04 | 0.2169 | 2.0117 | 0.9866 | 100 | 100 |
|  | **pseudouridine** | **1.63** | **0.0273** | 0.8755 | 1.423 | 100 | 100 | pseudouridine | -2.56 | 0.7244 | 3.1331 | 1.222 | 90 | 100 |
|  | **N4-acetylcytidine** | **1.59** | **0.0179** | 0.7866 | 1.2496 | 60 | 100 | N4-acetylcytidine |  |  |  |  |  |  |
|  | **cytidine** | **-1.92** | **0.0044** | 1.389 | 0.7183 | 100 | 100 | cytidine | 2.49 | 0.1199 | 0.9364 | 2.3316 | 90 | 100 |
|  | **5-methylcytidine** | **-1.45** | **0.0061** | 1.1864 | 0.8135 | 100 | 100 | 5-methylcytidine |  |  |  |  |  |  |
|  | **2'-deoxycytidine** | **-1.35** | **0.0049** | 1.077 | 0.797 | 100 | 50 | 2'-deoxycytidine | 3.46 | 0.5266 | 0.9962 | 3.4443 | 80 | 40 |
|  | thymine |  |  |  |  |  |  | **thymine** | **8.52** | **0.0358** | 0.4741 | 4.0371 | 30 | 70 |
|  | **3-aminoisobutyrate** | **3.1** | **0.0039** | 0.9125 | 2.829 | 100 | 100 | 3-aminoisobutyrate |  |  |  |  |  |  |
| Purine and Pyrimidine Metabolism | **methylphosphate** | **1.82** | **0.0082** | 0.7581 | 1.3783 | 100 | 90 | methylphosphate | -3.70 | 0.1542 | 3.0962 | 0.8504 | 90 | 100 |

| **Cholesterol and Bile Acids** | **Biochemical Name** | **Fold Change in Serum** | **Welch's Two-Sample *t*-Test** | **Mean Values** | | **% Filled Values** | | **Biochemical Name** | **Fold Change in Bile** | **Welch's Two-Sample *t*-Test** | **Mean Values** | | **% Filled Values** | |
| --- | --- | --- | --- | --- | --- | --- | --- | --- | --- | --- | --- | --- | --- | --- |
|  |  |  | **p-value** | **No GBM** | **GBM** | **No GBM** | **GBM** |  |  | **p-value** | **No GBM** | **GBM** | **No GBM** | **GBM** |
| Sterol | **lathosterol^12^** | **5.81** | **1.00E-04** | 0.4344 | 2.525 | 70 | 100 | lathosterol |  |  |  |  |  |  |
|  | **cholesterol^15^** | **1.56** | **5.00E-04** | 0.8853 | 1.3776 | 100 | 100 | cholesterol | -1.35 | 0.0652 | 1.1183 | 0.8301 | 100 | 100 |
|  | **7-alpha-hydroxycholesterol^7^** | **3.58** | **1.23E-05** | 0.4037 | 1.4471 | 70 | 100 | 7-alpha-hydroxycholesterol |  |  |  |  |  |  |
|  | **7-beta-hydroxycholesterol^19^** | **1.97** | **5.00E-04** | 0.7506 | 1.4763 | 100 | 100 | 7-beta-hydroxycholesterol |  |  |  |  |  |  |
|  | cholestanol |  |  |  |  |  |  | **cholestanol** | **-1.51** | **0.0135** | 1.0822 | 0.7112 | 80 | 0 |
|  | campesterol | 1.35 | 0.4544 | 0.8984 | 1.2131 | 100 | 90 | campesterol | -1.28 | 0.1209 | 1.1906 | 0.9274 | 100 | 100 |
|  | 7-alpha-hydroxy-3-oxo-4-cholestenoate (7-Hoca) | 1.37 | 0.2093 | 0.8115 | 1.1139 | 100 | 90 | 7-alpha-hydroxy-3-oxo-4-cholestenoate (7-Hoca) | |  |  |  |  |  |
|  | beta-sitosterol | 1.2 | 0.5262 | 0.667 | 0.7971 | 60 | 50 | beta-sitosterol |  |  |  |  |  |  |
| Steroid | cortisol | -1.61 | 0.3617 | 1.548 | 0.9582 | 90 | 100 | cortisol |  |  |  |  |  |  |
|  | cortisone | -1.09 | 0.7757 | 1.0415 | 0.9583 | 100 | 80 | cortisone |  |  |  |  |  |  |
|  | estriol 3-sulfate |  |  |  |  |  |  | estriol 3-sulfate | 4.91 | 0.3543 | 0.4381 | 2.1521 | 30 | 50 |
| Primary Bile Acid Metabolism | **cholate** | **20.18** | **0.0096** | 0.5555 | 11.2094 | 80 | 90 | cholate | 4.66 | 0.9915 | 1.1929 | 5.5606 | 70 | 60 |
|  | **taurocholate^17^** | **42.75** | **4.00E-04** | 0.4881 | 20.8671 | 100 | 100 | taurocholate**^27^** | -1.59 | 0.4016 | 1.153 | 0.7254 | 100 | 100 |
|  | **taurochenodeoxycholate^22^** | **44.42** | **0.0019** | 0.4917 | 21.8397 | 100 | 100 | taurochenodeoxycholate |  |  |  |  |  |  |
|  | **tauro(alpha + beta)muricholate** | **38.46** | **0.0325** | 0.5526 | 21.2526 | 70 | 70 | tauro(alpha + beta)muricholate |  |  |  |  |  |  |
|  | glycochenodeoxycholate |  |  |  |  |  |  | **glycochenodeoxycholate** | **-3.7** | **0.0352** | 3.4753 | 0.9337 | 90 | 60 |
|  | taurine |  |  |  |  |  |  | **taurine** | **-20.0** | **7.70E-05** | 2.9211 | 0.1495 | 90 | 90 |
|  | chenodeoxycholate |  |  |  |  |  |  | chenodeoxycholate | 11.50 | 0.3434 | 0.0187 | 0.215 | 0 | 20 |
| Secondary Bile Acid Metabolism | **taurodeoxycholate** | **67.0** | **0.0398** | 0.6739 | 45.1531 | 100 | 100 | taurodeoxycholate |  |  |  |  |  |  |
|  | glycodeoxycholate |  |  |  |  |  |  | **glycodeoxycholate** | **-8.33** | **0.0004** | 2.1929 | 0.2577 | 90 | 40 |
|  | taurolithocholate | 26.01 | 0.3107 | 0.844 | 21.9508 | 70 | 60 | **taurolithocholate^12^** | **-25.0** | **0.0003** | 5.306 | 0.2272 | 90 | 100 |
|  | **tauroursodeoxycholate^11^** | **45.16** | **0.0031** | 0.4024 | 18.1743 | 60 | 90 | tauroursodeoxycholate | 2.43 | 0.4614 | 1.1491 | 2.7947 | 100 | 100 |
|  | deoxycholate |  |  |  |  |  |  | deoxycholate | 2.94 | 0.2418 | 1.5527 | 4.5666 | 90 | 80 |

| **Lipids** | **Biochemical Name** | **Fold Change in Serum** | **Welch's Two-Sample *t*-Test** | **Mean Values** | | **% Filled Values** | | **Biochemical Name** | **Fold Change in Bile** | **Welch's Two-Sample *t*-Test** | **Mean Values** | | **% Filled Values** | |
| --- | --- | --- | --- | --- | --- | --- | --- | --- | --- | --- | --- | --- | --- | --- |
|  |  |  | **p-value** | **No GBM** | **GBM** | **No GBM** | **GBM** |  |  | **p-value** | **No GBM** | **GBM** | **No GBM** | **GBM** |
| Long Chain Fatty Acid | **margarate (17:0)** | **1.73** | **0.0181** | 0.6895 | 1.1931 | 100 | 100 | **margarate (17:0)** | **2.5** | **0.0276** | 0.9026 | 2.2605 | 100 | 100 |
|  | **10-heptadecenoate (17:1n7)** | **1.95** | **0.0117** | 0.753 | 1.4662 | 100 | 100 | 10-heptadecenoate (17:1n7) | 3.67 | 0.0627 | 1.3211 | 4.8513 | 100 | 100 |
|  | **erucate (22:1n9)** | **2.1** | **0.0406** | 0.8501 | 1.7888 | 100 | 100 | erucate (22:1n9) | 1.21 | 0.6994 | 0.9865 | 1.1934 | 100 | 100 |
|  | **pentadecanoate (15:0)^14^** | **1.97** | **0.0018** | 0.762 | 1.5027 | 100 | 100 | pentadecanoate (15:0) | -1.67 | 0.0773 | 1.3805 | 0.8221 | 100 | 100 |
|  | **cis-vaccenate (18:1n7)** | **1.77** | **0.0056** | 0.7245 | 1.2831 | 100 | 100 | **cis-vaccenate (18:1n7)** | **-2.13** | **0.0221** | 1.4661 | 0.6855 | 100 | 100 |
|  | oleate (18:1n9) | 1.27 | 0.1333 | 0.9669 | 1.2282 | 100 | 100 | **oleate (18:1n9)** | **-1.64** | **0.0479** | 1.1265 | 0.6862 | 100 | 100 |
| Polyunsaturated Fatty Acid (n3 and n6) | **linolenate [alpha or gamma; (18:3n3 or 6)]** | **2.83** | **0.0458** | 0.739 | 2.0945 | 100 | 100 | linolenate [alpha or gamma; (18:3n3 or 6)] | 1.92 | 0.1402 | 2.1223 | 4.0744 | 100 | 100 |
|  | stearidonate (18:4n3) | 6.13 | 0.0886 | 0.7821 | 4.7911 | 90 | 100 | stearidonate (18:4n3) | 5.02 | 0.1266 | 1.1827 | 5.9364 | 80 | 80 |
|  | **eicosapentaenoate (EPA; 20:5n3)** | **9.26** | **0.0242** | 0.9069 | 8.4024 | 100 | 100 | **eicosapentaenoate (EPA; 20:5n3)** | **13.66** | **0.0148** | 1.0601 | 14.4802 | 100 | 100 |
|  | **docosapentaenoate (n3 DPA; 22:5n3)** | **2.93** | **0.0054** | 0.7497 | 2.1985 | 100 | 100 | docosapentaenoate (n3 DPA; 22:5n3) | 6.6 | 0.0713 | 1.8918 | 12.4945 | 100 | 100 |
|  | **docosahexaenoate (DHA; 22:6n3)** | **4.32** | **0.0531** | 0.8942 | 3.8669 | 100 | 100 | **docosahexaenoate (DHA; 22:6n3)** | **11.2** | **0.0453** | 0.9487 | 10.6223 | 100 | 100 |
|  | linoleate (18:2n6) | 1.13 | 0.5244 | 1.0454 | 1.1857 | 100 | 100 | **linoleate (18:2n6)** | **2.63** | **0.0407** | 1.2236 | 3.2194 | 100 | 100 |
|  | arachidonate (20:4n6) | 1.04 | 0.9624 | 0.9794 | 1.0184 | 100 | 100 | **arachidonate (20:4n6)** | **2.55** | **0.0486** | 1.0088 | 2.5711 | 100 | 100 |
|  | dihomo-linolenate (20:3n3 or n6) | 1.13 | 0.7972 | 1.007 | 1.1379 | 100 | 100 | dihomo-linolenate (20:3n3 or n6) | 2.57 | 0.0901 | 1.4495 | 3.727 | 100 | 100 |
| Fatty Acid, Branched | 15-methylpalmitate (isobar with 2-methylpalmitate) | 1.82 | 0.0607 | 0.811 | 1.4743 | 100 | 100 | 15-methylpalmitate (isobar with 2-methylpalmitate) | 2.23 | 0.1353 | 0.9832 | 2.1877 | 100 | 100 |
| Fatty Acid, Dicarboxylate | octadecanedioate (stearic acid) | 4.18 | 0.0877 | 0.7858 | 3.2834 | 100 | 100 | octadecanedioate |  |  |  |  |  |  |
|  | **tetradecanedioate** | **-4.76** | **0.0011** | 2.4625 | 0.5131 | 100 | 70 | tetradecanedioate |  |  |  |  |  |  |
| Fatty Acid, Methyl Ester | stearate, methyl ester (methyl octadecanoate) | 1.16 | 0.1568 | 0.9457 | 1.0992 | 100 | 100 | **stearate, methyl ester^3^** | **3.2** | **3.02E-05** | 0.4883 | 1.5613 | 90 | 100 |
|  | linoleate, methyl ester | 1.43 | 0.3843 | 0.847 | 1.2095 | 80 | 100 | **linoleate, methyl ester** | **4.13** | **0.0077** | 0.4564 | 1.8869 | 20 | 80 |
| Fatty Acid, Amide | **stearamide^29^** | **1.66** | **0.0189** | 0.855 | 1.4208 | 100 | 100 | stearamide |  |  |  |  |  |  |
| Fatty Acid, Amino | **2-aminoheptanoate** | **-2.22** | **0.0343** | 1.5846 | 0.713 | 100 | 90 | 2-aminoheptanoate |  |  |  |  |  |  |
| Fatty Acid Metabolism (also BCAA Metabolism) | **butyrylcarnitine** | **2.03** | **0.0163** | 0.6792 | 1.3799 | 90 | 100 | **butyrylcarnitine^5^** | **-2.94** | **0.0001** | 1.9643 | 0.6655 | 100 | 100 |
|  | propionylcarnitine | 0.96 | 0.6161 | 1.1304 | 1.082 | 100 | 100 | **propionylcarnitine** | **-2.32** | **0.0184** | 1.8897 | 0.8209 | 100 | 100 |
| Fatty Acid Metabolism(Acyl Carnitine) | **hexanoylcarnitine** | **1.71** | **0.0284** | 0.7149 | 1.2193 | 90 | 100 | hexanoylcarnitine |  |  |  |  |  |  |
|  | octanoylcarnitine | 1.71 | 0.0648 | 0.8442 | 1.4467 | 70 | 100 | octanoylcarnitine |  |  |  |  |  |  |
|  | acetylcarnitine | 1.53 | 0.0636 | 0.9077 | 1.3879 | 100 | 100 | acetylcarnitine | 0.86 | 0.7894 | 1.292 | 1.1142 | 100 | 100 |
| Carnitine Metabolism | **deoxycarnitine** | **-1.37** | **0.0495** | 1.1365 | 0.8272 | 100 | 100 | deoxycarnitine |  |  |  |  |  |  |
|  | **3-dehydrocarnitine** | **-1.85** | **0.0505** | 1.6931 | 0.9211 | 100 | 100 | 3-dehydrocarnitine | 0.66 | 0.5484 | 1.754 | 1.1596 | 90 | 100 |
| Ketone Bodies | acetoacetate |  |  |  |  |  |  | **acetoacetate** | **3.88** | **0.0427** | 0.9088 | 3.5283 | 100 | 100 |
| Fatty Acid, Monohydroxy | 2-hydroxystearate | 0.88 | 0.4525 | 1.1229 | 0.9861 | 100 | 100 | 2-hydroxystearate | 4.15 | 0.0532 | 0.9318 | 3.8651 | 100 | 100 |
| Eicosanoid | thromboxane B2 | -1.2 | 0.0926 | 1.2613 | 1.0466 | 100 | 90 | thromboxane B2 |  |  |  |  |  |  |
| Endocannabinoid | **N-stearoyltaurine** | **2.1** | **0.0124** | 0.7262 | 1.5258 | 80 | 90 | **N-stearoyltaurine** | **-2.04** | **0.0196** | 1.3752 | 0.6747 | 90 | 100 |
|  | N-oleoyltaurine | 1.63 | 0.641 | 1.0962 | 1.7868 | 100 | 100 | **N-oleoyltaurine^17^** | **-3.57** | **0.0102** | 2.026 | 0.5656 | 100 | 100 |
|  | N-palmitoyltaurine | 1.27 | 0.678 | 1.1079 | 1.4055 | 100 | 80 | **N-palmitoyltaurine** | **-2.94** | **0.0171** | 1.7469 | 0.5925 | 100 | 100 |
| Inositol Metabolism | **chiro-inositol** | **3.92** | **0.0075** | 0.6271 | 2.4581 | 80 | 90 | chiro-inositol |  |  |  |  |  |  |
|  | **pinitol** | **2.56** | **0.025** | 0.7045 | 1.8015 | 50 | 80 | pinitol |  |  |  |  |  |  |
|  | **scyllo-inositol** | **2.53** | **0.0174** | 0.9684 | 2.4481 | 100 | 100 | scyllo-inositol | 2.04 | 0.1585 | 1.0188 | 2.0753 | 80 | 90 |
|  | **myo-inositol** | **-1.35** | **0.002** | 1.2129 | 0.9007 | 100 | 100 | myo-inositol | 0.83 | 0.9253 | 1.3844 | 1.1531 | 100 | 100 |
| Phospholipid Metabolism | **choline^20^** | **1.63** | **3.62E-05** | 0.7834 | 1.275 | 100 | 100 | choline | 1.01 | 0.7477 | 1.2905 | 1.3086 | 100 | 100 |
|  | glycerophosphorylcholine (GPC) | 1.13 | 0.7006 | 0.9114 | 1.0308 | 100 | 100 | glycerophosphorylcholine (GPC) | 1.42 | 0.099 | 0.879 | 1.2474 | 90 | 100 |
|  | phosphoethanolamine |  |  |  |  |  |  | **phosphoethanolamine** | **-2.04** | **0.0011** | 1.1531 | 0.5643 | 90 | 30 |
| Lysolipid | **1-eicosapentaenoylglycerophosphocholine (20:5n3)** | **9.56** | **0.0041** | 0.6943 | 6.6359 | 100 | 100 | **1-eicosapentaenoylglycerophosphocholine (20:5n3)** | **6.9** | **0.0175** | 0.6131 | 4.2282 | 70 | 90 |
|  | 1-margaroylglycerophosphocholine (17:0) | 0.92 | 0.9391 | 1.2688 | 1.1654 | 90 | 90 | **1-margaroylglycerophosphocholine (17:0)** | **5.16** | **0.0463** | 0.841 | 4.3399 | 90 | 100 |
|  | 1-linolenoylglycerophosphocholine (18:3n3) | 3.1 | 0.1605 | 0.9639 | 2.9915 | 80 | 70 | 1-linolenoylglycerophosphocholine (18:3n3) | 9.39 | 0.0637 | 0.6886 | 6.4659 | 80 | 100 |
|  | 2-palmitoylglycerophosphocholine | 0.77 | 0.7204 | 1.7368 | 1.3376 | 100 | 100 | 2-palmitoylglycerophosphocholine | 4.76 | 0.0597 | 0.9883 | 4.7015 | 100 | 100 |
|  | 2-oleoylglycerophosphocholine | 1.43 | 0.8432 | 1.544 | 2.2033 | 100 | 100 | 2-oleoylglycerophosphocholine | -4.35 | 0.0822 | 5.3844 | 1.2421 | 100 | 100 |
|  | 1-nonadecanoylglycerophosphocholine(19:0) | -2.0 | 0.0941 | 1.2658 | 0.6359 | 60 | 40 | 1-nonadecanoylglycerophosphocholine(19:0) | |  |  |  |  |  |
|  | **1-margaroylglycerophosphoethanolamine** | **2.67** | **0.0015** | 0.6951 | 1.8587 | 100 | 100 | 1-margaroylglycerophosphoethanolamine | 7.15 | 0.0593 | 0.8532 | 6.1035 | 90 | 100 |
|  | **2-oleoylglycerophosphoethanolamine** | **2.5** | **0.0179** | 0.6691 | 1.6722 | 80 | 100 | 2-oleoylglycerophosphoethanolamine | 7.83 | 0.0716 | 0.7363 | 5.7638 | 60 | 90 |
|  | **1-oleoylglycerophosphoethanolamine** | **2.45** | **0.0201** | 0.817 | 2.0024 | 100 | 100 | 1-oleoylglycerophosphoethanolamine | 7.47 | 0.0588 | 0.9109 | 6.8068 | 100 | 100 |
|  | **1-linoleoylglycerophosphoethanolamine** | **2.28** | **0.012** | 0.7763 | 1.7684 | 100 | 100 | **1-linoleoylglycerophosphoethanolamine** | **3.63** | **0.0176** | 0.7788 | 2.83 | 100 | 100 |
|  | **1-stearoylglycerophosphoethanolamine** | **2.03** | **0.0018** | 0.8606 | 1.748 | 100 | 100 | **1-stearoylglycerophosphoethanolamine** | **6.42** | **0.0494** | 0.9787 | 6.2829 | 100 | 100 |
|  | 1-arachidonoylglycerophosphoethanolamine | 1.71 | 0.0678 | 0.8969 | 1.537 | 100 | 100 | 1-arachidonoylglycerophosphoethanolamine | 2.04 | 0.1171 | 0.8938 | 1.8246 | 90 | 100 |
|  | 1-stearoylplasmenylethanolamine | 1.36 | 0.6872 | 1.0096 | 1.3745 | 100 | 100 | **1-stearoylplasmenylethanolamine** | **9.87** | **0.0151** | 0.6517 | 6.4321 | 80 | 100 |
|  | 2-palmitoylglycerophosphoethanolamine | 1.55 | 0.2234 | 0.9094 | 1.4059 | 100 | 100 | 2-palmitoylglycerophosphoethanolamine | 5.25 | 0.0551 | 0.8256 | 4.3357 | 90 | 90 |
|  | 1-palmitoylglycerophosphoethanolamine | 1.49 | 0.3476 | 1.0507 | 1.5693 | 100 | 100 | 1-palmitoylglycerophosphoethanolamine | 5.08 | 0.0804 | 0.9554 | 4.856 | 100 | 100 |
|  | 1-stearoylglycerophosphoserine | 3.26 | 0.5132 | 1.3137 | 4.2876 | 90 | 80 | **1-stearoylglycerophosphoserine^7^** | **102.9** | **0.0065** | 0.3403 | 35.0185 | 30 | 100 |
|  | 1-arachidonoylglyercophosphate | 1.48 | 0.0886 | 0.939 | 1.3864 | 100 | 100 | 1-arachidonoylglyercophosphate |  |  |  |  |  |  |
|  | 2-oleoylglycerophosphoglycerol |  |  |  |  |  |  | **2-oleoylglycerophosphoglycerol** | **-6.25** | **0.0014** | 2.7602 | 0.4298 | 90 | 80 |
|  | 1-oleoylglycerophosphoglycerol |  |  |  |  |  |  | **1-oleoylglycerophosphoglycerol** | **-1.85** | **0.0413** | 1.2842 | 0.689 | 90 | 100 |
| Glycerolipid Metabolism | **glycerol** | **1.48** | **0.0271** | 0.8882 | 1.3141 | 100 | 100 | glycerol | -1.85 | 0.0615 | 1.6674 | 0.8929 | 100 | 100 |
|  | glycerol 3-phosphate (G3P) | 1.28 | 0.1369 | 0.9758 | 1.2509 | 100 | 100 | glycerol 3-phosphate (G3P) | 1.98 | 0.0511 | 0.7174 | 1.4221 | 100 | 100 |
| Monoacylglycerol | **1-linoleoylglycerol (1-monolinolein)** | **1.42** | **0.0389** | 0.7706 | 1.0951 | 100 | 100 | 1-linoleoylglycerol (1-monolinolein) | 3.12 | 0.1149 | 1.6722 | 5.2181 | 100 | 100 |
|  | 1-docosahexaenoylglycerol (1-monodocosahexaenoin) |  |  |  |  |  |  | 1-docosahexaenoylglycerol (1-monodocosahexaenoin) | 7.23 | 0.085 | 0.8157 | 5.8972 | 80 | 80 |
| Diacylglycerol | **1,3-dipalmitoylglycerol** | **2.83** | **0.0453** | 0.7079 | 2.0057 | 100 | 100 | **1,3-dipalmitoylglycerol^24^** | **-3.57** | **0.0001** | 1.4648 | 0.4109 | 90 | 60 |
|  | **1,2-dipalmitoylglycerol** | **2.55** | **0.0064** | 0.8618 | 2.201 | 100 | 100 | **1,2-dipalmitoylglycerol^22^** | **-3.57** | **0.0002** | 1.5533 | 0.4404 | 90 | 40 |
| Sphingolipid Metabolism | **palmitoyl sphingomyelin** | **1.56** | **0.0435** | 0.8235 | 1.2827 | 100 | 100 | palmitoyl sphingomyelin |  |  |  |  |  |  |
|  | **stearoyl sphingomyelin^30^** | **2.05** | **0.0004** | 0.7837 | 1.6062 | 100 | 100 | stearoyl sphingomyelin | 1.12 | 0.6691 | 0.9999 | 1.1164 | 100 | 100 |

| **Xenobiotics** | **Biochemical Name** | **Fold Change in Serum** | **Welch's Two-Sample *t*-Test** | **Mean Values** | | **% Filled Values** | | **Biochemical Name** | **Fold Change in Bile** | **Welch's Two-Sample *t*-Test** | **Mean Values** | | **% Filled Values** | |
| --- | --- | --- | --- | --- | --- | --- | --- | --- | --- | --- | --- | --- | --- | --- |
|  |  |  | **p-value** | **No GBM** | **GBM** | **No GBM** | **GBM** |  |  | **p-value** | **No GBM** | **GBM** | **No GBM** | **GBM** |
| Benzoate Metabolism | **methyl-4-hydroxybenzoate** | **1.75** | **0.0132** | 0.8939 | 1.5631 | 100 | 100 | **methyl-4-hydroxybenzoate** | **4.01** | **0.0464** | 0.8276 | 3.3182 | 100 | 100 |
|  | hippurate | 1.01 | 0.8482 | 1.1133 | 1.1192 | 40 | 30 | hippurate | 6.52 | 0.0653 | 0.891 | 5.8127 | 100 | 100 |
|  | benzyl alcohol |  |  |  |  |  |  | **benzyl alcohol^11^** | **-9.1** | **8.17E-05** | 1.0558 | 0.1146 | 100 | 10 |
|  | catechol sulfate | 0.34 | 0.1191 | 2.2929 | 0.7705 | 80 | 50 | **catechol sulfate** | **-5.0** | **0.0117** | 1.1983 | 0.2338 | 70 | 50 |
|  | **benzoate** | **-1.35** | **0.0151** | 1.2633 | 0.9403 | 100 | 100 | benzoate | 0.55 | 0.6133 | 1.6954 | 0.9317 | 100 | 100 |
|  | 4-hydroxyhippurate |  |  |  |  |  |  | 4-hydroxyhippurate | -5.0 | 0.0844 | 3.4189 | 0.6893 | 80 | 40 |
| Food component | equol sulfate | -2.08 | 0.1998 | 2.369 | 1.131 | 80 | 20 | **equol sulfate^15^** | **-33.3** | **0.0008** | 6.396 | 0.2064 | 90 | 100 |
|  | daidzein |  |  |  |  |  |  | **daidzein** | **-5.26** | **0.0388** | 5.4536 | 1.0288 | 100 | 100 |
|  | genistein |  |  |  |  |  |  | genistein | -5.88 | 0.0769 | 3.9801 | 0.6899 | 90 | 70 |
|  | **homostachydrine** | **-6.25** | **0.0197** | 3.2076 | 0.5121 | 80 | 70 | **homostachydrine** | **-4.76** | **0.0074** | 1.7536 | 0.3718 | 90 | 60 |
|  | **stachydrine** | **-2.38** | **0.0044** | 1.4593 | 0.6122 | 100 | 100 | **stachydrine** | **-3.12** | **0.0059** | 2.1323 | 0.6722 | 100 | 100 |
|  | 3-hydroxycinnamate (m-coumarate) |  |  |  |  |  |  | **3-hydroxycinnamate (m-coumarate)** | **-5.88** | **0.019** | 0.9484 | 0.1576 | 70 | 0 |
|  | 5-ketogluconate |  |  |  |  |  |  | **5-ketogluconate** | **-1.45** | **0.0491** | 1.5168 | 1.0418 | 100 | 100 |
|  | **tartarate^24^** | **-6.67** | **0.0004** | 1.0133 | 0.1498 | 90 | 30 | tartarate |  |  |  |  |  |  |
| Hemoglobin and Porphyrin Metabolism | heme | 6.19 | 0.3447 | 1.6643 | 10.2973 | 90 | 100 | **heme** | **10.52** | **0.006** | 0.4927 | 5.1825 | 90 | 100 |
|  | D-urobilin |  |  |  |  |  |  | D-urobilin | 24.52 | 0.052 | 0.2608 | 6.394 | 30 | 50 |
| Drug | pentobarbital | 0.92 | 0.3434 | 0.76 | 0.6999 | 20 | 0 | **pentobarbital^8^** | **-9.10** | **8.93E-05** | 1.1896 | 0.1263 | 100 | 0 |
|  | hydroquinone sulfate | 0.57 | 0.1017 | 1.4 | 0.7966 | 100 | 100 | hydroquinone sulfate | -1.51 | 0.0622 | 1.4341 | 0.9525 | 100 | 100 |
| Chemical | **diisopropanolamine** | **1.71** | **0.0149** | 0.4834 | 0.8274 | 20 | 80 | **diisopropanolamine^21^** | **-7.69** | **0.0002** | 2.7912 | 0.3615 | 100 | 50 |
|  | S-(3-hydroxypropyl)mercapturic acid (HPMA) | 1.04 | 0.7432 | 1.0942 | 1.1354 | 100 | 100 | **S-(3-hydroxypropyl)mercapturic acid (HPMA)^18^** | **-4.17** | **2.00E-05** | 2.4023 | 0.5707 | 100 | 70 |
|  | 3-hydroxypyridine |  |  |  |  |  |  | 3-hydroxypyridine | -2.08 | 0.0695 | 2.6034 | 1.254 | 100 | 100 |
